# Spatiotemporal Variation in Pollination Deficits in an Insect-Pollinated Dioecious Crop

**DOI:** 10.3390/plants10071273

**Published:** 2021-06-22

**Authors:** Helena Castro, Catarina Siopa, Vinícius Casais, Mariana Castro, João Loureiro, Hugo Gaspar, Maria Celeste Dias, Sílvia Castro

**Affiliations:** Centre for Functional Ecology, Department of Life Sciences, University of Coimbra, Calçada Martim de Freitas, 3000-456 Coimbra, Portugal; catarinasiopa@gmail.com (C.S.); viniciuscasais@gmail.com (V.C.); mcastro@uc.pt (M.C.); jloureiro@bot.uc.pt (J.L.); hugo.gaspar.1997@gmail.com (H.G.); celeste.dias@uc.pt (M.C.D.); scastro@bot.uc.pt (S.C.)

**Keywords:** *Actinidia*, ecosystem services, hand-pollination, Kiwifruit, pollen limitation, production

## Abstract

Inadequate quantity and quality of pollen reaching the stigmas decreases the sexual reproductive output of plants, compromising yield. Still, the current extent of pollen limitation affecting yield (i.e., pollination deficits) is poorly quantified. This study is aimed at quantifying pollination deficits in kiwifruit orchards, a dioecious plant with a fruit caliber and market value largely dependent on pollination services. For that, we set up a pollination experiment and quantified services and yield provided by current pollination vectors, and under optimal pollination, over two years in a total of twenty-three orchards covering the kiwifruit production range in Portugal. We characterized nine fruit traits and used: (1) fruit weight to calculate pollination deficits and relate them with pollinator diversity and abundance, and environmental variables; and (2) production values, fruit caliber, and market values to calculate economic impact of pollination deficits. Results showed that pollination deficits were variable in time and space and were significantly and negatively correlated with pollinator abundance, while the opposite pattern was obtained for production, supporting the notion that a higher pollinator’s abundance is related to lower pollination deficits and higher yields. Understanding the factors affecting pollination deficits is crucial to depict the need for nature-based solutions promoting pollinators and to resort to management practices assisting pollination.

## 1. Introduction

The yield and quality of over 75% of crops worldwide is directly affected by animal pollination [1], and the area occupied by pollinator dependent crops has increased over the last decades [2]. Pollination is therefore an important biodiversity-dependent service supporting food provisioning. Pollination is the simple transfer of pollen from the anthers to the stigmas, culminating in fertilization. However, it is far from being simple, as plants rely on mutualistic interactions with animals to carry their pollen, frequently suffering pollen limitation [3]. Pollination deficit can be defined as the inadequate quantity and quality of pollen reaching the stigmas, which decreases the sexual reproductive output of plants [4]. It can result from factors such as insufficient or inefficient pollen transfer due to limited pollinator abundance and diversity, low pollinator activity or efficiency, and/or flowering asynchrony [4]. In this context, pollinators’ decline as a result of major global changes such as land use changes (e.g., fragmentation and agriculture intensification), pesticide use, biological invasions, and eutrophication constitutes a threat to pollination services supporting food production [4,5]. Pollination deficits in crops have been assessed indirectly though the quantification of pollinator populations [4,6,7], but direct estimates of pollination deficits and their impact on crop productivity are scarcer (see, however, [8,9,10]). Because pollination deficits are the direct result of pollinator activity and available pollinator populations, quantifying pollinator diversity and abundance and relating it with crop yield is crucial to understanding the observed pollination deficits. Additionally, the direct estimates of pollination deficits measure the effect of limited pollination services on reproductive output from a plant perspective (fitness) and, if linked with productivity, it may also consider the farmer’s perspective, which is more focused on agronomic and economic yields [6].

Kiwifruit (*Actinidia* spp., Actinidiaceae) is a dioecious crop mainly pollinated by insects, with buzz pollinators being pointed out as more efficient [11] and, to a lesser extent, by wind [12,13]. Kiwifruit caliber and market value are largely influenced by the number of seeds formed, which in turn depend on the number and quality of pollen grains reaching the stigmas [14]. Therefore, efficient pollination is a key aspect in kiwifruit production and its economic viability. High quality market fruits (>100 g) have over 1000 seeds, and to ensure good pollination each flower should receive an estimated number of 2000–3000 viable pollen grains [12,15,16]. In addition, kiwifruit pollination is highly dependent on weather conditions during flowering time, which often limit insect pollination (e.g., [12,17,18]). Finally, the short flowering period, the lack of synchrony between male and female plants, and the low number and/or inadequate distribution of males within the orchard further contribute to an inefficient pollination [19]. While wind pollination can contribute to pollination, enabling the attainment of a high fruit set, its effectiveness is considered highly inconsistent and insufficient [12,13]. As a result of these hurdles, kiwifruit producers frequently include management practices such as the installation of honeybee colonies or the application of artificial pollen [16,20]. However, these practices have two drawbacks. First, honeybees are not the most efficient pollinator and are more sensitive to poor weather conditions when compared to wild pollinators [11,21]. Second, purchased pollen has a high cost, and contributes to the dissemination of diseases, such as the *Pseudomonas syringae pv. actinidiae*, during pollen application [20,22].

In key kiwifruit production areas worldwide (e.g., New Zealand and Italy) there are well-established practices and guidelines for producers (e.g., [23,24,25]). In these areas, artificial pollen application is a common and cost-effective practice, as pollination resources are scarce due to the vast plantation areas [25]. However, the reality is different in countries with smaller but still significant contribution to the kiwifruit worldwide market, such as Portugal and Spain. In Portugal, kiwifruit was introduced in 1973, the production started in the 1980’s [26], and the production area has grown steadily over the last decades (currently representing around 2736 ha). This region is characterized by small-scale orchards in a mosaic landscape [27]. Landscapes composed of different mosaics of land-uses may provide multiple resources for pollinator populations and promote wild pollinator communities [28,29]. Consequently, pollination requirements may vary significantly from those in massive plantations, setting different challenges for management practices. However, and despite the current knowledge of the limitations to an efficient pollination, the magnitude of pollination deficits in kiwifruit orchards (and in many other crops), its relation with available pollinator communities, and subsequent economic impact are completely unknown. Such information is important to assist decision making and optimize crop production.

Our study aims to quantify pollination services in kiwifruit orchards representing the entire production area of Portugal in order to provide real estimates on pollination deficits, relate them with available pollinator communities, and assess potential impacts on kiwifruit production and market value. We hypothesize that: (1) wind has a significant contribution to fruit set, but it is insufficient to attain high quality market fruits; (2) if current pollination services are inadequate, fruit set and fruit caliber from supplemented flowers will be higher than those from open pollinated flowers, and this will be reflected on overall orchard production and monetary gain; and (3) pollination services, and hence the levels of pollination deficits, are influenced by pollinator diversity and abundance and environmental variables which may explain temporal and spatial variability. To test our hypotheses, we set up a pollination experiment over two years in a total of twenty-three orchards and quantified nine fruit traits and pollination deficits. Environmental and pollinator variables were used to explain pollination deficits and production. Finally, production values, fruit caliber, and market values were used to calculate economic impact of pollination deficits.

## 2. Results

### 2.1. Contribution of Wind to Kiwifruit Pollination

Wind pollination produced variable results in fruit set with overall non-significant differences between open and wind pollination and a significantly lower fruit set when compared to supplementary pollination (Table 1). Within an orchard, wind pollination resulted in significantly lower fruit set when compared to supplementary pollination or to both open and supplementary pollination in three of the orchards, while no differences were detected in the remaining orchards and varieties (Table S1).

**Figure 1:**
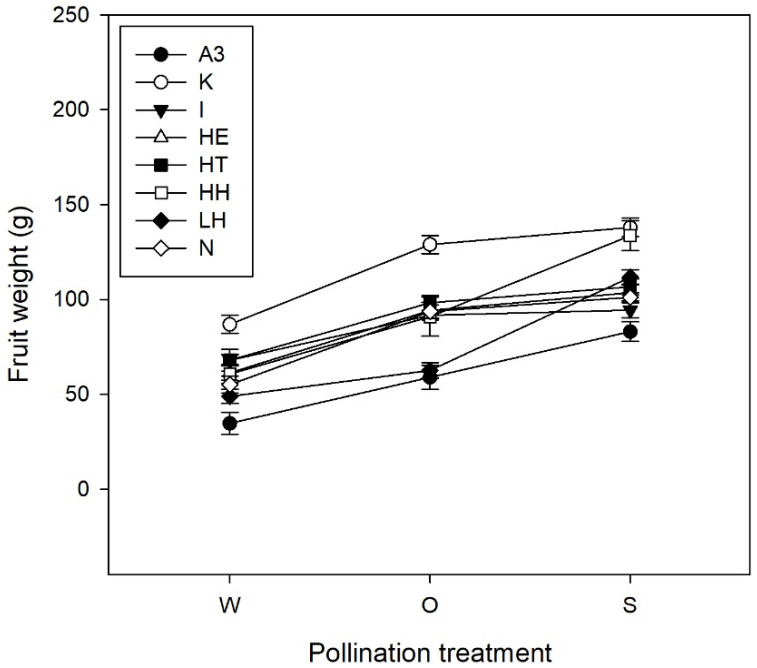
Mean fruit weight (g) ± SE of kiwifruits resulting from wind (W), open (O) and supplementary (S) pollination treatments applied in the nine orchards surveyed in 2018.

Overall, wind pollination produced small, nonmarketable fruits with weights significantly lower when compared to open and supplementary pollination (Table 1; Figure 1; Table S2). A significant effect of wind pollination was also obtained on dry matter, flesh firmness, acidity, S:L, and TSS (Table 1). Wind pollinated fruits showed, on average, lower dry matter content when compared to supplementary pollination, lower acidity and TSS when compared to open pollination, lower flesh firmness when compared to both open and supplementary pollination, and higher S:L when compared to supplementary pollination (Table 1). Within orchards, non-significant differences among treatments were observed for most fruit variables (*p* > 0.05; Table S2).

### 2.2. Pollination Deficits

Fruit set differed significantly between pollination treatments within year, while no differences were observed between years (Table 2). Fruit set was higher in supplemented flowers compared to open pollinated ones (Table 2). Within orchards and variety, fruit set of open pollinated flowers was significantly higher than that of supplementary pollinated flowers in five orchards (one in 2018 and four in 2019; Table S1).

Fruit weight varied significantly between pollination treatments within year, while no differences were observed between years (Table 2). Overall, fruits resulting from supplementary pollination were heavier than those resulting from open pollination (Table 2). Within orchards and varieties, a similar pattern was observed. Supplemented flowers produced heavier fruits than the ones produced by open pollinated flowers, although this was significant only in eight orchards/varieties (two in 2018 and six in 2019; Tables S2 and S3). For the remaining fruit variables, dry matter, flesh firmness, and S:L differed significantly between years, and none of these fruit variables showed variation between pollination treatment within a year (Table 2). Within the orchards, we found significant differences only for flesh firmness in two orchards and for S:L in one orchard in 2019 (Tables S2 and S3).

**Figure 2:**
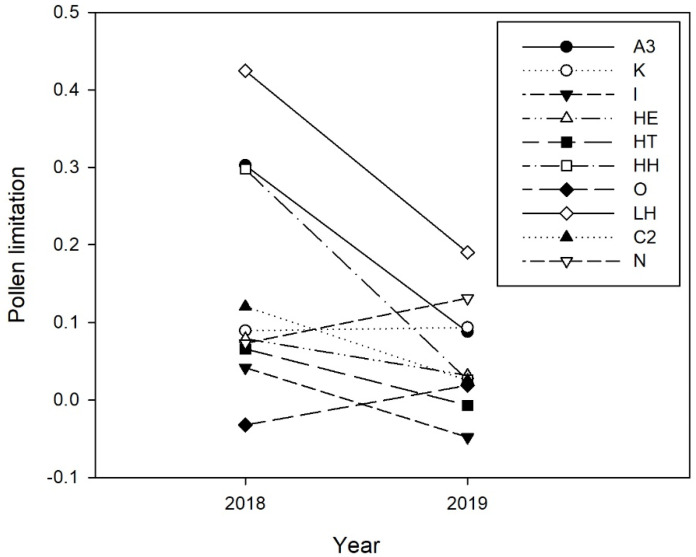
Yearly variation in pollination deficit in the orchards surveyed both in 2018 and 2019.

**Figure 3:**
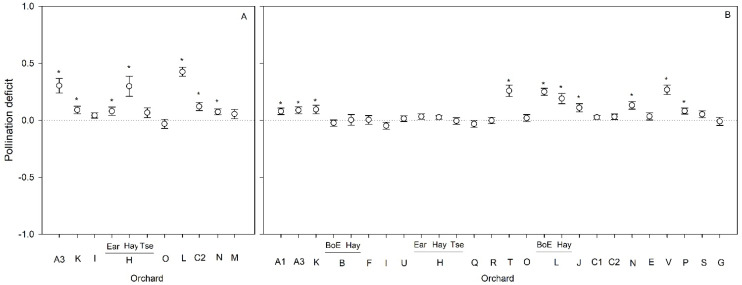
Pollination deficit (mean ± SE) in the nine orchards surveyed in 2018 (**A**) and in the twenty-two orchards surveyed in 2019 (**B**). Statistically significant differences (*p* < 0.05) in pollination deficit resulting from one-sample *t*-test analyses are indicated by an asterisk.

Overall, there was a significant decrease in pollination deficits in 2019 when compared to 2018 (Table 2, Figure 2). Within year, we found significant pollination deficits in six orchards out of nine in 2018 (Figure 3A). In 2019, we found pollination deficits in ten orchards out of twenty-two (Figure 3B). In both years, pollination deficits varied among orchards, from −0.03 to 0.43 in 2018, and from −0.05 to 0.27 in 2019.

### 2.3. Linking Pollination Deficits and Production with Environmental and Pollinator’s Related Variables

Regression analysis showed a significant negative relation between honeybee abundance and pollination deficit (Table 3), indicating that higher abundances of honeybees were associated with lower pollination deficit. Additionally, a significant and positive relation was obtained between the abundance of honeybees and *Bombus* species and productivity, indicating that higher abundances of these pollinators were associated with higher productivity levels. In contrast, a significant negative relation was found between pollinator richness and productivity, indicating an opposite pattern (Table 3). The two environmental variables included in the regression analyses showed non-significant relations with both pollination deficit and production (Table 3).

### 2.4. Linking Pollination Deficit with Production and Market Value

Data show high variability in fruit weight (Tables S2 and S3) within orchards. Consequently, fruits were distributed over several caliber categories (Figure S1). A significant effect of pollination treatment within years on the proportion of small and large fruits was observed, while no differences were observed between years (Table 2). Supplementary pollination resulted in a lower proportion of small fruits and a higher proportion of large fruits in both years when compared to open pollination (Table 2). When analyzed individually, orchards that showed pollination deficit also showed clear and similar patterns as the ones described above for the percentage of fruit per caliber category (Table S3). Overall, supplementary pollinated flowers produced a significantly (*p* < 0.05) or marginally significant (*p* < 0.10) lower proportion of small fruits compared to open pollination in three out of nine orchards in 2018, and in six out of twenty-two in 2019 (Tables S2 and S3). Supplementary pollination produced significantly (*p* < 0.05) or marginally significant (*p* < 0.10) higher proportion of large fruits compared to open pollination in four orchards out of nine in 2018, in five orchards out of twenty-two in 2019 (Tables S2 and S3).

**Figure 4:**
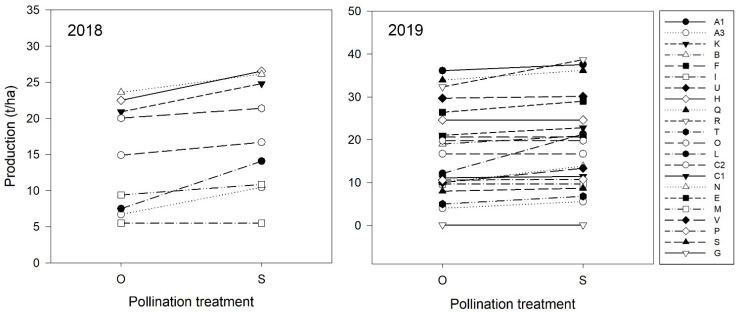
Kiwifruit production (t/ha) corresponding to current orchard pollination services (i.e., open pollination, O) and estimated production under optimal pollination services (i.e., supplementary pollination, S) for the nine orchards surveyed in 2018 (**left**) and the twenty-two orchards surveyed in 2019 (**right**).

Overall, kiwifruit production was not significantly affected by year, although there was a tendency toward an increase in production in 2019 when compared to 2018 (Table 2). Significant differences were observed between pollination treatments within years (Table 2), with supplementary pollination contributing to higher production values than open pollination in 2018 and 2019 (Table 2). Data further support that production can be increased by adequate pollen supply (Figure 4), particularly in orchards showing pollination deficit as was the case of orchard L in 2018, where a clear increase in production resulting from supplementary pollination can be observed (Figure 4).

Our calculations revealed that differences in the percentage of small and large fruits, resulting from adequate pollen supply (Tables S2 and S3), can translate into monetary gains (Figure S2). The monetary gain was not significantly affected by year, although there is a tendency toward an increase in monetary gain in 2019 when compared to 2018 (Table 2). Significant differences were observed between pollination treatments within years (Table 2), with an increase in the amount of euros under supplementary when compared to open pollination. When analyzed individually, the pattern is clear for several orchards in both years (Figure S2).

## 3. Discussion

Like many worldwide crops, efficient pollination is a key aspect in kiwifruit production and its economic viability [1,16,18]. In particular, kiwifruit is a dioecious plant that needs high pollen loads (which are transported by biotic and abiotic vectors) to attain marketable fruits [14,16]. This study provides the first spatio-temporal quantification of pollination deficits in kiwifruit orchards across a wide production area. The study region is characterized by productive systems with features that are very different from those of the high kiwifruit yield systems in leading countries, where most studies were performed and where most methodologies have been optimized (e.g., [23,24,25]; but see [18,19] in Spain). Overall, our results show an inefficient pollination by wind and variation in both space and time in pollination services, although significant levels of pollination deficits were observed only in some orchards and/or years. The increased abundance of main pollinator groups (i.e., honeybee and *Bombus*) were significantly related to a decreasing pollination deficit and increasing production levels. Moreover, pollen supply significantly improved the production in several orchards and, consequently, may result in increased economic revenue to kiwifruit producers. The study, while being mostly descriptive and focused on kiwifruit, is of global interest, provides information that is applicable to other crops, and opens way for further works exploring orchard and landscape factors affecting pollination services to crops. Below, we discuss the results in detail, linking with orchard management practices.

### 3.1. Contribution of Wind to Kiwifruit Pollination

Kiwifruit flowers have two main pollination syndromes: (1) wind pollination marked by pendulous flowers with large and fleshy stigmas, production of high quantities of pollen, and synchronous mass flower production lasting a short period of time [24,30]; and (2) insect-pollination, specifically buzz pollination, marked by the production of attractive flowers, floral scent, high amounts of pollen as reward, gradually maturing anthers, and a large number of ovules [11,30,31,32]. Although representing one of the two pollination syndromes in kiwifruit, our results show that wind pollination is not sufficient to guarantee adequate pollination for commercial production. The results are consistent with other studies, which reported that, while wind contributes to kiwifruit pollination, it is highly inconsistent and insufficient to attain high quality market fruits (e.g., [12,13,18]). Kiwifruit pollen is dry and carried in the air, and the use of large fans to blow pollen from male flowers and maximize wind pollination is a common practice used by producers in leading kiwifruit producing countries such as Italy [24]. In Portugal, this practice is not common and fans were used in five orchards only. In one of the orchards that used fans, wind pollination produced only 22.7% of fruits in the large and medium fruit categories against 83.9% resulting from open pollination, suggesting that this management practice is still insufficient to attain successful pollination levels. Altogether, the results obtained here highlight the importance of entomophilous pollination to attain marketable kiwifruits.

### 3.2. Spatio-Temporal Variation of Pollination Deficit

Sampling kiwifruit orchards in two consecutive years and doing so over a wide area of the production range allowed us to observe the existence of both spatial and temporal variation in pollination deficits. Pollination deficits can result from the lack of pollen and/or inefficient pollen transport, both of which can be affected by the available pollinator communities, management factors, and/or weather conditions [18,19,33,34]. While precipitation and temperature did not have a significant effect, the abundance of the main pollinator’s groups was negatively correlated with pollination deficits and positively correlated with orchard production, supporting that variation in pollinator communities is one of the factors driving productivity. In particular, increased abundances of honeybees led to lower pollination deficits, while both honeybee and *Bombus* species abundances contributed to higher productivity in kiwifruit orchards. Honeybees and *Bombus* species are indeed the main pollinators in kiwifruit orchards, with honeybees usually attaining high densities which may compensate for their lower kiwifruit pollination efficiencies in comparison to *Bombus* species [11,18]. Miñarro and Twizell [18] observed that honeybees were more abundant and visited more kiwifruit flowers per unit of time, but *Bombus* species were more efficient on a per visit basis, which resulted in an overall non-significant effect of pollinator species on fruit weight. Contrary to other studies that show increased pollination services with increased pollinator richness (e.g., [35]), in our case pollinator richness was negatively related to kiwifruit production. This may be explained by: (1) the efficiency of individual pollinator species, as demonstrated by MacInnis and Forrest [36] for strawberries, who found that bee identity had the strongest effect on yield; and (2) by the low abundance of other pollinators [27], which results in these having lower contribution to kiwifruit pollination and production regardless of their contribution to overall pollinator’s richness. Thus, in the case of kiwifruit, management practices and landscape contexts that promote efficient pollinator groups will likely promote pollination effectiveness and productivity.

Interestingly, for a significant number of orchards in both years, we did not find significant differences in fruit weight between open and supplementary pollination, indicating that the available pollinator communities were enough to ensure adequate pollination for commercial production. When the orchards have a good provision of pollination services (either through the wild pollinator communities alone or by its complementation with *Apis mellifera* colonies) and of pollen (through an adequate male to female ratio, proper distribution of males and flowering synchronization), natural pollination levels can be sufficient to produce high quality marketable fruits. Several studies have shown that pollination services are affected by the interplay between landscape composition and heterogeneity and farm management, and that landscape simplification and intensive farm management have a synergistically negative effect on pollination services [37,38,39,40,41]. In Portugal, kiwifruit orchards are of a small size, with low pesticide input and low insect non-friendly management practices, and are imbedded in a diversified landscape [27]. These factors may contribute to the maintenance of wild pollinator communities in the studied orchards, contributing to the successful pollination of kiwifruit. Future studies should explore the in-field and landscape factors determining pollination services in kiwifruit orchards in heterogenous landscapes such as the ones observed in our study region.

As mentioned above, regularly maintained management practices that impact insects, such as pesticide use, vegetation management, or hail net coverage steadily affect pollinator communities and their behavior [19,42,43]. Four orchards showed pollination deficits in both years and were signaled by the producers as regularly having problems with pollination (author’s field observations). These orchards deserve a thorough evaluation of the management practices that may be impacting pollination services, as it can be one of the main factors affecting productivity. For instance, two of these orchards are covered by a hail net, which is an important factor affecting pollination, as these structures reduce ventilation and pollen movement and restrict movements from pollinators and other beneficial insects and can change or reduce the visual cues necessary for their orientation [20,43]. Also, in New Zealand, a high number of bees were observed foraging uncovered orchards, with flowers in these orchards receiving more visits when compared to the visitation rate observed in covered kiwifruits orchards [43]. Additionally, orchard A3, was very affected by the bacterial disease *Pseudomonas syringae pv. Actinidiae*, which is an important disease in kiwifruit and contributes to pollination deficit through significant impacts in male flowering and pollen availability, with negative consequences in kiwifruit production [20].

### 3.3. Pollination Services, Production and Market Value

In our study, pollen supplementation tended to increase productivity and the economic value of kiwifruits, either by increasing total fruit production (evident in several orchards in 2018) or by increasing fruit caliber (evident in several orchards in 2019), both affecting the total economic revenue for the producer. This was more evident in orchards that showed pollination deficits, further highlighting the importance of adequate pollination for kiwifruit production. The importance of pollination for kiwifruit weight and size, which determine economic values, is well documented and has been demonstrated in several studies (e.g., [13,18,44,45]), although none was made at such a large scale. Also, and despite its importance, pollination is still considered the least understood management factor in kiwifruit orchards [24]. A better understanding of the pollination needs at a local scale and across large geographic areas, as we did here, is thus fundamental to improve production and to a more efficient use of resources, which might ultimately result in higher profit for kiwifruit producers.

The results presented in this study reflect a maximum potential gain following optimal pollination. Future studies should incorporate a cost-benefit analysis that considers not only the cost of the various management practices but also potential benefits to pollination services. As observed in our study, it is also important to consider that pollination deficits may vary in space and time due to variable pollinator communities, which may also be a determinant of the management practices needed to improve pollination in some orchards (e.g., the need for artificial pollination). Nevertheless, it is clear that kiwifruit production, and hence producers’ monetary gain, can be increased by adequate pollination. Natural pollination could potentially be enhanced by the use of insect friendly practices such as increasing wild bee nesting sites, the maintenance of diverse hedgerow, or the implementation of green infrastructures as food supplies before and after the crop flowering period [40,46,47]. Additionally, producers may consider complementing wild pollinator’s community with managed bee colonies or, ultimately, and in very particular cases (due to the costs associated and other problems), complementing natural pollination with artificial pollen application [16,20].

## 4. Materials and Methods

### 4.1. Studied Orchards

The pollination experiments were conducted in 9 orchards in the spring of 2018 and in 22 orchards in the spring of 2019, 8 of which were surveyed in both years (Table S4). The orchards are located in the North and Centre of Portugal (Figure S3). All but one orchard (orchard G with organic farming) were conventionally managed. Nineteen orchards were planted with *Actinidia deliciosa* and three orchards were planted with *A. chinensis* (both dioecious species). Details of kiwifruit orchards and kiwifruit male and female varieties used in this study are given in Table S4. For each orchard locality, environmental variables were extracted from climate data obtained from IPMA (Portuguese Institute for Sea and Atmosphere). Environmental variables extracted include precipitation during the flowering period, because of its effect on pollen movement and insect activity, and average mean temperature in the 1.5 months before flowering peak because of its effect on flowering synchrony and duration [33].

In the study region, the flowering period generally starts in mid-May, but in 2018 the flowering was delayed and occurred in late May–early June. In 2019, flowering occurred in the second half of May but showed high heterogeneity among orchards. The most abundant pollinators found in kiwifruit orchards are *Apis mellifera*, followed by *Bombus* spp., and more rarely other wild bees (e.g., *Halictus* spp.) and Syrphidae (e.g., *Eristalis*, *Melanostoma* and *Sphaerophoria* species; [18,27]). Pollinator richness and abundance was shown to vary among the 22 sampled orchards [27]. A parallel study focused on pollinator community was undertaken by [27] during 2019 at the same 22 orchards samples here. Thus, data on overall pollinator richness and abundance, and on the abundance of the two main pollinator groups, honeybees and *Bombus*, were taken from [27] and covers the 22 orchards sampled in 2019.

### 4.2. Pollination Experiments

To quantify pollination deficits in kiwifruit orchards we set up a pollination experiment in the flowering seasons between 2018 and 2019, with the following pollination treatments: (1) open pollination (O), i.e., unmanipulated flowers left open for natural levels of pollination, thereby enabling to quantify the services provided by the available pollinator community; and (2) supplementary pollination (S), i.e., flowers left open to natural levels of pollination and pollinated with fresh pollen from up to eight male flowers of the varieties available in the orchard, thereby enabling to quantify yield under optimal pollination services. In 2018, the following additional treatment was conducted to quantify the contribution of wind: (3) emasculated open pollination (W), i.e., flowers in which petals and anthers were removed at the bud stage and left open, thereby enabling to quantify the services provided by wind. Preliminary pollinator visitation observations revealed that removing anthers and petals (insect attractants) prevented insect visitation to emasculated flowers (author’s field observations). Based on the consistent results obtained in 2018, on the reduced contribution of wind reported in the literature (e.g., [12,13]) and on the increased number of fields surveyed in 2019, in the second year we decided to exclude the evaluation of wind contribution. In detail, for each orchard and variety, at peak of orchard flowering, we selected 30 female plants separated by at least 3 m, in a line at the middle of the orchard, in most cases covering the entire length of the orchard. Per plant, 2–3 flowers, depending on whether two or three pollination treatment were applied, located in the same branch, to guarantee similar resource availability, were marked and assigned to a given treatment. Receptive flowers with petals starting to fall and sticky stigmatic surfaces [16] were selected for both open and supplementary pollinations. Supplementary pollinations were performed by gently rubbing anthers of up to eight freshly collected male flowers with dehiscent pollen (visible by whitish anther pores and visible clouds of pollen when rubbing the flowers). Male flowers were collected from male varieties present in the orchard.

### 4.3. Fruit Processing

The collection of kiwifruits was coordinated with each orchard producer so that fruits could be collected as close as possible to the harvest date. Thus, fruits were collected, on average two days before the harvesting of the orchard. At harvest time, the fruits from marked flowers were collected and the percentage of flowers that set fruit was calculated (fruit set). All fruits resulting from the flowers marked for pollination treatments were weighed, and measured for short (at the equatorial zone) and long (apical-basal axis) diameters with a digital caliper; all deformations and skin damages were also registered. Additionally, we determined flesh firmness, °Brix, acidity (n = 10 and all fruits per treatment in 2018 and 2019, respectively), and dry matter content (n = 10 and 15 fruits per treatment in 2018 and 2019, respectively). Flesh firmness was measured by puncture with a handheld digital penetrometer (Fruit hardness tester, STEPS Systems) fitted with a flat 8 mm diameter plunger after removing 1 mm skin at the fruit equator. °Brix was measured with a hand-held digital refractometer (Kern Optics), and acidity was measured with an ATAGO PAL-Easy ACID F5 by taking a juice sample from longitudinally cut fruit half of each fruit. For acidity, the juice sample was diluted in distilled water (1:50) according to the manufactures’ instructions. The second half of each fruit was used to determine dry matter content, recording the fresh weight and the dry weight after drying at 60 °C until achieving a constant weight. Dry matter content was given by: (dry weight/fresh weight) * 100 and is expressed in percentage.

In 2018, the fruit half used for °Brix and acidity was also used to determine total soluble sugars (TSS), starch and total antioxidant capacity (TAA). The samples were triturated with a blender and three subsamples of 10 mg were weighed, placed in extraction buffer, and stored at −20 °C until analysis. Total soluble sugars were quantified following Irigoyen et al. [48]. For this, one subsample per fruit was homogenized with ethanol (80%, *v*/*v*) and incubated at 80 °C for 1 h; after centrifugation at 5000× *g* for 10 min at 4 °C, one aliquot of the supernatant was mixed with an anthrone solution (40 mg anthrone + 1 mL dH_2_O + 20 mL H_2_SO_4_) and incubated for 10 min at 100 °C; samples were cooled and centrifuged as described above. Starch was quantified according to Osaki et al. [49]. For this, another subsample was homogenized with perchloric acid (30%, *v*/*v*) and incubated at 60 °C for 1 h. After centrifugation at 10,000× *g* for 10 min at 4 °C, one aliquot of the supernatant was incubated with the anthrone solution (as described for TSS), followed by another centrifugation (5000× *g* for 10 min and 4 °C). For both TSS and starch, the absorbance of the supernatant was read at 625 nm using a Multilabel Reader (EnSpire™, PerkinElmer, Norwalk, USA) and the contents calculated using the standard curve of glucose (y = 5.1061x + 0.2279, r^2^ = 0.9995, for TSS and y = 1.0514x + 0.2719, r^2^ = 0.9919 for starch). For TAA, the last subsample was homogenized with 1 mL of methanol and incubated at 40 °C for 30 min. The mixture was centrifuged at 15,000× *g* for 15 min at 4 °C and one aliquot of the supernatant was mixed with ABTS [2,20-azino-bis (3-ethylbenzothiazoline-6-sulphonic acid)]. The absorbance was read at 734 nm (Re et al., 1999) and TAA was calculated using a gallic acid standard curve (y = 1094.5x − 38.977, r^2^ = 0.9855).

### 4.4. Pollination Deficit

Pollination deficit was calculated using the commonly used formula to calculate pollen limitation, and was obtained for each plant based on fruit weight, according to Larson and Barrett [50]: PL = 1 − O/S, where O is the fruit weight of the open pollination treatment, and S is the fruit weight of the supplementary pollination treatment. Positive values resulting from higher fruit weight in supplementary versus open pollination treatments indicate pollination deficit and, consequently, the occurrence of pollination deficits, while zero or negative values indicate no pollination deficit. In cases where we obtained negative values for pollination deficit, we considered that these indicate the absence of pollination deficit and were treated as zero in subsequent calculations in this study. Despite our attempts to minimize them, negative values may result from within individual differences (e.g., ovary size, resource allocation, shadow) known to affect fruit weight within plants [51,52,53].

### 4.5. Commercial Grading

Commercial grading of every sampled kiwifruit was assigned following standard grading tables provided by the Portuguese Association of Kiwifruit producers (APK—Associação Portuguesa de Kiwicultores). The tables assign fruits into classes and calibers. First, kiwifruits were assigned to class I or class II based on their short to long diameter ratio (S:L), deformation, and skin damages. Fruits with an S:L below 0.75, deformed or bearing skin damage, were assigned to class II, while fruits with an S:L equal or above 0.75, well-formed and without skin damages, were assigned to class I. Secondly, within classes, fruits were assigned to one of 11 caliber categories (namely, 18, 20, 23, 25, 27, 30, 33, 36, 39, 42, or 46 category) based on their weight (each category representing the average number of fruits of that size to attain 1 kg of kiwifruit). Fruits with weights lower than 65 g were considered with no market value.

### 4.6. Economic Analysis

We estimated effects of pollination deficit on kiwifruit production by comparing yields under natural (open pollination) and optimal pollination (supplementary pollination). Orchard production values of 2018 and 2019 were provided by each kiwifruit producer and taken as the values corresponding to production under natural pollination. Production under optimal pollination was estimated, taking into account the gain in fruit set in supplementary pollinated fruits compared to natural pollination and the pollination deficit values obtained for each orchard according to the following equation: Popt = Po + (Po * PL) + (Po * Fs), where Popt is the production under optimal pollination, Po is the production under open pollination, PL is the pollination deficit, and Fs is the difference in fruit set between supplementary and open pollination treatments.

We estimated the economic effects of pollination deficit by comparing the monetary gain under open and optimal pollination. Monetary gain was obtained by calculating the amount, in Euros, corresponding to each orchard production, both under open and supplementary pollination, considering the percentage of fruits in each class and caliber, and the corresponding monetary value. Average prices paid to the producer according to class and caliber assignments were provided by APK. Calculations were done according to the following equation: Monetary gain (euros) = ∑ P * Ci * Ei, where *P* is the production (*t*/ha) of a given field under natural or optimal pollination (calculated as described above), Ci is the proportion of fruits in each class and caliber combination, and Ei is the price, in Euros, payed per kg of fruit for each class and caliber combination in a given year. These were then used to calculate the difference in monetary gain resulting from supplementary pollination.

### 4.7. Statistical Analyses

Generalized linear models (GLM) were used to explore differences between pollination treatments within orchards on fruit parameters (namely, fruit set, weight, dry matter, flesh firmness, °Brix, acidity, S:L, TSS, starch, TAA, and fruit caliber). Fruit weight and quality parameters were analyzed using a Gaussian distribution with the identity link function, and fruit set and fruit distribution by caliber categories were analyzed using a binomial distribution with the logit link function. For the GLM analysis, fruit caliber was grouped into three categories, adapted from NZKGI [25]: (1) large fruits, which are fruits with high market quality and includes calibers 18 to 30; (2) medium fruits, which includes calibers 33 to 36; and (3) small fruits, which includes fruits in calibers 39, 42, 46, fruits in class II, and non-marketable fruits. The effect of pollination treatment on fruit set and fruit parameters across orchards was analyzed using generalized linear mix models (GLMM) with pollination treatment as a fixed factor and orchard and kiwifruit variety as random factors. The S:L was transformed using arcsin (sqrt) and the TAA was transformed using sqrt. The effect of year and pollination treatment within year on fruit set, fruit parameters, and pollination deficit, production and monetary gain across orchards was analyzed using GLMM with year and pollination treatment nested within year as fixed factors, and orchard, kiwifruit variety, precipitation, and average mean temperature as random factors. Model validation was performed on the residuals by checking heteroscedasticity and normality [54] and response variables were transformed when necessary to achieve such requirements. One-sample *t*-test was used to test if pollination deficits within orchard differed significantly from zero. Regression models using pollinator richness, honeybee abundance, *Bombus* spp. abundance, precipitation, and temperature as predictors were used to explain patterns in pollination deficits and production across orchards. Only orchards for which pollinator variables and production were obtained in the same year were included in this analysis. Other pollinators’ related variables (e.g., overall pollinator abundance, wild pollinator richness and abundance) showed significant correlations and, thus, were excluded from the regression analyses.

All analyses were done using R version 3.3.2 [55] using the package “car” for Type-III analysis of variance [56] and “lme4” for generalized linear models and generalized linear mixed models [57], and “multcomp” for multiple comparisons after Type-III analysis of variance in GLMM [58] and TukeyHSD for multiple comparisons after analysis of variance in GLM.

## 5. Conclusions

The quantification of pollination deficits across the production area of Portugal enabled us to conclude that pollination deficits are variable both in time and space, likely resulting from a combination of multiple factors that determine pollen availability and pollinator’s communities. Although pollen supplementation increased productivity and the economic values of kiwifruits, positively affecting the producer’s economic revenue, in general, a representative number of kiwifruit orchards showed low pollination deficits, suggesting sufficient pollination services. Still, consistent pollination deficits in some orchards suggest the existence of factors that consistently affect pollination success. Consequently, future studies should address how orchard management practices and landscape factors may affect kiwifruit production, contributing with relevant data to design guidelines towards productive and sustainable agroecosystems.

## Figures and Tables

**Table 1:** Generalized mixed-effect model analyses of the effect of pollination treatments (O, Open; S, Supplementary; W, Wind) on fruit set and fruit parameters (S:L, fruit short to long diameter ratio; TSS, total soluble sugars; TAA, total antioxidant capacity and starch). Statistically significant differences are indicated by different letters and highlighted in bold.

Variable	Means ± SE			*X*^2^ or *F*	*p* Values
	Wind	Open	Supplement		
Fruit set (%)	**66.52 ± 3.18 (b)**	**73.20 ± 2.81 (b)**	**85.60 ± 2.22 (a)**	***X*^2^_2_ = 24.72**	**<0.001**
Weight (g)	**61.82 ± 2.02 (c)**	**89.76 ± 2.01 (b)**	**107.17 ± 1.87 (a)**	***F*_2, 527.6_ = 164.51**	**<0.001**
Dry matter (%)	**14.75 ± 0.28 (b)**	**15.14 ± 0.21 (a, b)**	**15.66 ± 0.21 (a)**	***F*_2, 214.0_ = 164.51**	**<0.001**
Flesh firmness (N)	**40.59 ± 2.08 (b)**	**42.74 ± 1.85 (a)**	**43.96 ± 1.75 (a)**	***F*_2, 215.03_ = 6.12**	**0.003**
°Brix (%)	6.03 ± 0.23	6.12 ± 0.20	6.21 ± 0.20	*F*_2, 215.0_ = 0.246	0.783
Acidity (%)	**1.46 ± 0.03 (b)**	**1.55 ± 0.03 (a)**	**1.50 ± 0.03 (a, b)**	***F*_2, 208.25_ = 3.175**	**0.044**
S:L	**0.88 ± 0.00 (a)**	**0.87 ± 0.00 (a, b)**	**0.86 ± 0.00 (b)**	***F*_2, 515.9_ = 3.08**	**0.047**
TAA (µmol eq. galic acid/g)	3.08 ± 0.12	3.07 ± 0.10	2.98 ± 0.10	*F*_2, 211.0_ = 0.354	0.702
TSS (mg/g)	**4.81 ± 0.18 (b)**	**5.33 ± 0.18 (a)**	**5.31 ± 0.19 (a, b)**	***F*_2, 201.2_ = 3.61**	**0.029**
Starch (mg/g)	2.89 ± 0.06	2.93 ± 0.04	2.90 ± 0.05	*F*_2, 204.3_ = 0.118	0.889

**Table 2:** Generalized mixed-effect model analyses of the effect of year or the effects of year and pollination treatment (Open vs. Supplementary pollination) nested within year on pollination deficit, fruit set and fruit parameters (S:L, fruit short to long diameter ratio). Values for year and pollination treatments are given as means and SE of the mean. Statistically significant differences are highlighted in bold.

Factors	Year				Year: Pollination Treatment					
Variables	2018	2019	*X*^2^ or *F*	*p* Values	2018		2019		*X*^2^ or *F*	*p* Values
					Open	Supplement	Open	Supplement		
Fruit set (%)	78.68 ± 1.57	85.94 ± 0.88	*X*^2^_1_ = 1.51	0.219	**73.24 ± 2.40**	**84.12 ± 1.99**	**82.99 ± 1.34**	**88.92 ± 1.13**	***X*^2^_2_ = 26.27**	**<0.001**
Weight (g)	97.95 ± 1.22	103.65 ± 0.75	*F*_1, 20.26_ = 0.256	0.619	**91.89 ± 1.61**	**104.56 ± 1.53**	**100.57 ± 1.08**	**106.67 ± 1.02**	***F*_2, 1865_ =44.30**	**<0.001**
Dry matter (%)	**15.56 ± 0.14**	**14.03 ± 0.07**	***F*_1, 20.0_ = 0.063**	**<0.001**	15.45 ± 0.19	15.88 ± 0.19	14.00 ± 0.10	14.07 ± 0.10	*F*_2, 1140_ = 2.72	0.066
Flesh firmness (N)	**47.24 ± 0.90**	**70.38 ± 0.35**	***F*_1, 7.9_ =65.65**	**<0.001**	46.89 ± 1.30	47.57 ± 1.24	70.14 ± 0.78	70.62 ± 1.24	*F*_2, 1519_ = 1.26	0.284
°Brix (%)	6.19 ± 0.12	6.56± 0.05	*F*_1, 19.0_ = 1.00	0.331	6.34 ± 0.16	6.26 ± 0.16	6.42 ± 0.07	6.57 ± 0.07	*F*_2, 1479_ = 0.21	0.814
Acidity (%)	1.52 ± 0.02	1.54 ± 0.01	*F*_1, 12.4_ = 0.13	0.728	1.54 ± 0.03	1.51 ± 0.02	1.54 ± 0.01	1.55 ±0.01	*F*_2, 1511_= 0.69	0.501
S:L	**0.88 ± 0.00**	**0.90 ± 0.00**	***F*_1, 10.6_ = 11.52**	**0.006**	0.88 ± 0.00	0.90 ± 0.00	0.88 ± 0.00	0.90 ± 0.00	*F*_2, 1789_= 1.29	0.274
Pollination deficit	**0.11 ± 0.01**	**0.06 ± 0.01**	***F*_1, 8.4_ = 5.47**	**0.046**	-	-	-	-	-	-
Large fruits (%)	50.98 ± 2.11	58.35 ± 1.34	*X*^2^_1_ = 0.35	0.554	**42.86 ± 2.89**	**59.93 ± 3.00**	**54.45 ± 1.92**	**62.19 ± 1.85**	***X*^2^_2_ = 29.33**	**<0.001**
Medium fruits (%)	24.06 ± 1.81	20.53 ± 1.10	*X*^2^_1_ = 1.17	0.282	24.49 ± 2.51	23.60 ± 2.60	20.18 ± 1.55	20.88 ± 1.55	*X*^2^_2_ = 0.21	0.901
Small fruits (%)	24.96 ± 1.83	21.12 ± 1.11	*X*^2^_1_ = 0.67	0.412	**32.65 ± 2.74**	**16.48 ± 2.27**	**25.37 ± 1.68**	**16.93 ± 1.43**	***X*^2^_2_ = 38.40**	**<0.001**
Production (t/ha)	15.97 ± 1.76	18.07 ± 1.63	*F*_1, 7.3_ = 0.07	0.801	**14.56 ± 2.46**	**17.38 ± 2.57**	**17.18 ± 2.27**	**18.97 ± 2.38**	***F*_1, 28_ = 11.71**	**<0.001**
Monetary gain (€/ha)	14,842.25± 1820.73	18,479.98± 2105.59	*F*_1, 7.3_ = 0.01	0.948	**12,385.04** **± 2912.32**	**17,299.47** **± 2664.70**	**16,935.04** **± 2340.69**	**20,024.43** **± 3075.26**	***F*_1, 28_ = 7.35**	**0.003**

**Table 3:** Regression analyses of the effect of environmental (precipitation and temperature) and pollinator’s related variables (pollinator richness, honeybee abundance, and *Bombus* spp. abundance) on pollination deficit and production. Statistically significant differences are highlighted in bold.

Variables	Pollination Deficit			Production		
	Estimate	*t* Value	*p*	Estimate	*t* Value	*p*
Pollinator richness	0.0007	0.267	0.792	**−0.603**	**−2.600**	**0.021**
Honeybee abundance	**−0.0004**	**−2.286**	**0.034**	**0.039**	**2.399**	**0.031**
*Bombus* spp. abundance	−0.0003	−0.822	0.421	**0.077**	**2.206**	**0.045**
Precipitation	0.0005	0.502	0.622	0.106	1.274	0.224
Temperature	0.0186	0.698	0.494	−2.086	−0.726	0.480

## Data Availability

Data are available from the authors upon request.

## Supplementary Materials

The following are available online at https://www.mdpi.com/article/10.3390/plants10071273/s1, Table S1: Fruit set; Tables S2 and S3: Fruit weight and quality parameters in 2018 and 2019, respectively; Figure S1: Distribution of kiwifruits by caliber category; Figure S2: Change in monetary gain; Table S4: Details of kiwifruit orchards; Figure S3: Distribution of sampling orchards.
