# Supplementary material for: Spatiotemporal Variation in Pollination Deficits in an Insect-Pollinated Dioecious Crop
Source: Plants (Basel). 2021 Jun 22;10(7):1273. doi: 10.3390/plants10071273 (PMC8309125; doi:10.3390/plants10071273)
Supplement: Supplementary file 1 [file plants-10-01273-s001.zip › plants-1257305-supplementary.pdf]

Table S1. Fruit set (%) from wind, open and supplementary pollinated kiwifruit flowers in the 9 orchards surveyed in 2018 (left) and the 22 orchards surveyed in 2019. X^2 and *P* values resulting from Generalized linear model analysis are shown. Statistically significant results are highlighted in bold and differences among pollination treatments are indicated by different letters.

Orchard	Variety	2018				2019		
		Wind	Open	Supplement	Statistical test	Open	Supplement	Statistical test
A1	BoErica	-	-	-	-	100.00	100.00	$X^2_{1, 58}$ = 0.00 *P* =1.000
A3	*A. chinensis*	**51.61(b)**	**54.84(ab)**	**80.65(a)**	**$X^2_{2, 90}$ = 7.09 *P* = 0.029**	**30.00(b)**	**66.67(a)**	**$X^2_{2, 58}$ = 8.27 *P* = 0.004**
B	BoErica	-	-	-	-	96.77	96.77	$X^2_{1, 60}$ = 0.00 *P* = 1.000
	Hayward	-	-	-	-	93.33	83.33	$X^2_{1, 58}$ = 1.50 *P* = 0.221
C1	Hayward	-	-	-	-	100.00	100.00	$X^2_{1, 58}$ = 0.00 *P* = 1.000
C2	Hayward	-	86.67	86.67	$X^2_{1, 58}$ = 0.00 *P* = 1.000	100.00	93.33	$X^2_{1, 58}$ = 2.84 *P* = 0.092
E	*A. chinensis*	-	-	-	-	80.65	87.10	$X^2_{1, 60}$ = 0.48 *P* = 0.489
F	Earlygreen	-	-	-	-	90.00	86.67	$X^2_{1, 58}$ = 0.16 *P* = 0.687
G	BoErica	-	-	-	-	76.67	76.67	$X^2_{1, 58}$ = 0.00 *P* = 1.000
H	Earlygreen	83.33	90.00	86.67	$X^2_{2, 87}$ = 0.58 *P* = 0.747	100.00	96.67	$X^2_{1, 58}$ = 1.40 *P* = 0.236
	Tsechelidis	66.67	80.00	86.67	$X^2_{2, 84}$ = 3.37 *P* = 0.186	93.33	90.00	$X^2_{1, 58}$ = 0.22 *P* =0.639
	Hayward	81.25	87.50	93.75	$X^2_{2, 93}$ = 3.06 *P* = 0.217	96.67	96.67	$X^2_{1, 58}$ = 0.00 *P* = 1.000
I	Hayward	88.57	85.71	80.00	$X^2_{2, 102}$ = 1.02 *P* = 0.601	93.33	93.33	$X^2_{1, 58}$ = 0.00 *P* = 1.000
J	Hayward	-	-	-	-	23.81	36.36	$X^2_{1, 41}$ = 0.81 *P* = 0.369
K	BoErica	**48.39(b)**	**74.19(ab)**	**83.87(a)**	**$X^2_{2, 90}$ = 9.69 *P* = 0.008**	93.33	93.33	$X^2_{1, 58}$ = 0.00 *P* = 1.000
L	BoErica	-	-	-	-	**53.33(b)**	**96.67(a)**	**$X^2_{1, 58}$ = 17.26 *P* < 0.001**
	Hayward	**66.67(ab)**	**45.16(b)**	**90.32(a)**	**$X^2_{2, 71}$ = 15.58 *P* < 0.001**	**36.67(b)**	**90.00(a)**	**$X^2_{1, 58}$ = 19.92 *P* < *0.001***
M	Hayward	-	73.33	83.33	$X^2_{1, 58}$ = 0.89 *P* = 0.345	-	-	
N	Hayward	**39.13(b)**	**90.00(a)**	**93.33(a)**	**$X^2_{2, 80}$ = 24.31 *P* < 0.001**	86.49	80.00	$X^2_{1, 65}$ = 0.51 *P* = 0.477
O	Hayward	-	60.00	70.00	$X^2_{1, 58}$ = 0.66 *P* = 0.416	90.63	87.50	$X^2_{1, 62}$ = 0.16 *P* = 0.688
P	Hayward	-	-	-	-	96.67	90.00	$X^2_{1, 58}$ = 1.12 *P* = 0.290
Q	Hayward	-	-	-	-	**80.00(b)**	**100.00(a)**	**$X^2_{1, 58}$ = 8.99 *P* = 0.003**
R	Soreli	-	-	-	-	80.00	90.00	$X^2_{1, 58}$ = 1.20 *P* = 0.274

Table S1. (Cont.)

Orchard	Variety	2018				2019		
		Wind	Open	Supplement	Statistical test	Open	Supplement	Statistical test
S	Hayward	-	-	-	-	90.00	93.33	$X^2_{1, 58} = 0.22$ $P = 0.639$
T	Hayward	-	-	-	-	73.33	83.33	$X^2_{1, 58} = 0.89$ $P = 0.345$
U	Hayward	-	-	-	-	93.33	93.33	$X^2_{1, 58} = 0.00$ $P = 1.000$
V	Hayward	-	-	-	-	90.00	96.67	$X^2_{1, 58} = 1.12$ $P = 0.290$

Table S2. Mean values (± std error) of weight and quality parameters of kiwifruits resulting from wind, open and supplementary pollination treatments applied in the 9 orchards surveyed in 2018. *F or* X^2 and *P* values resulting from Generalized linear model analysis are shown. Statistical significant differences are highlighted in bold and differences among pollination treatments resulting from Tukey post-hoc test are indicated by different letters. Large fruits include fruits within caliber 18 to 30, Medium include fruits within caliber 33 to 36 and Small fruits include fruits in calibers 39 to 46, class II and non-marketable fruits. 39 to 46, Class II and non-marketable fruits.

Orchard	Variety	Pollination treatment	Weight (g)	Dry matter (%)	Flesh firmness (N)	ºBrix	Acidity	S:L	TAA	TSS	Starch	Large fruits (%)	Medium fruits (%)	Small fruits (%)
A3	*A. chinensis*	Wind	**34.67 ±5.82(c)**	14.01 ±0.45	15.66 ±1.66	6.50 ±0.45	1.57 ±0.12	0.90 ±0.01	3.54 ±0.19	**5.07 ±0.13(b)**	2.94 ±0.08	**5.55(b)**	0.00	94.44
		Open	**58.95 ±6.32(b)**	14.75 ±0.36	17.11 ±1.66	7.49 ±0.51	1.91 ±0.06	0.89 ±0.01	3.99 ±0.24	**5.66 ±0.29(ab)**	2.95 ±0.08	**18.52(ab)**	18.52	62.96
		Supplement	**83.05 ±5.16(a)**	15.47 ±0.35	18.01 ±1.59	7.31 ±0.49	1.69 ±0.10	0.88 ±0.01	3.67 ±0.15	**6.10 ±0.28(a)**	3.01 ±0.04	**42.31(a)**	19.23	38.46
			**$F_{2, 68}$ = 15.38**<br>***P* < 0.001**	$F_{2, 24}$ = 3.36<br>*P* = 0.051	$F_{2, 24}$ = 0.51<br>*P* = 0.609	$F_{2, 24}$ = 0.94<br>*P* = 0.404	$F_{2, 24}$ = 1.86<br>*P* = 0.058	$F_{2, 67}$ = 2.23<br>*P* = 0.115	$F_{2, 24}$ = 1.34<br>*P* = 0.282	**$F_{2, 22}$ = 3.74**<br>***P* = 0.040**	$F_{2, 24}$ = 0.43<br>*P* = 0.654	**$X^2_{2, 68}$ = 9.14**<br>**p = 0.010**	**$X^2_{2, 68}$ = 6.39**<br>**p = 0.041**	$X^2_{2, 68}$ = 6.39<br>p = 0.041
C2	Hayward	Open	94.69 ±4.18	15.27 ±0.47	55.57 ±1.50	7.08 ±0.58	1.38 ±0.07	0.88 ±0.01	1.65 ±0.11	4.76 ±0.29	3.00 ±0.03	44.83	31.03	24.14
		Supplement	105.61 ±4.47	14.60 ±0.56	57.12 ±1.79	7.27 ±0.53	1.47 ±0.07	0.88 ±0.01	1.79 ±0.07	5.26 ±0.38	3.01 ±0.02	69.23	11.54	19.23
			$F_{1, 52}$ = 3.19<br>*P* = 0.080	$F_{1, 52}$ = 0.81<br>*P* = 0.379	$F_{1, 17}$ = 0.43<br>*P* = 0.522	$F_{1, 18}$ = 0.06<br>*P* = 0.813	$F_{1, 17}$ = 2.83<br>*P* = 0.111	$F_{1, 50}$ = 0.21<br>*P* = 0.999	$F_{1, 18}$ = 1.14<br>*P* = 0.301	$F_{1, 18}$ = 1.08<br>*P* = 0.312	$F_{1, 17}$ = 0.25<br>*P* = 0.625	$X^2_{1, 53}$ = 3.37<br>*P* = 0.067	$X^2_{1, 53}$ = 3.19<br>*P* = 0.074	$X^2_{1, 53}$ = 0.19<br>*P* = 0.659
H	Earlygreen	Wind	**61.31 ±3.99(b)**	13.24 ±0.25	**31.70 ±2.90(b)**	8.54 ±0.30	1.35 ±0.09	0.85 ±0.01	3.05 ±0.15	6.32 ±0.36	2.72 ±0.22	**8.00(b)**	4.00	**88.00(a)**
		Open	**94.18 ±4.25(a)**	13.72 ±0.27	**40.10 ±1.88(a)**	8.16 ±0.26	1.44 ±0.08	0.80 ±0.02	2.90 ±0.10	6.43 ±0.54	2.52 ±0.18	**40.74(a)**	25.93	**33.33(b)**
		Supplement	**103.57 ±4.13(a)**	14.18 ±0.35	**42.00 ±1.60(a)**	8.17 ±0.36	1.52 ±0.10	0.81 ±0.02	2.97 ±0.11	6.86 ±0.57	2.18 ±0.26	**57.69(a)**	19.23	**23.08(b)**
			**$F_{2, 75}$ = 28.16**<br>***P* < 0.001**	$F_{2, 26}$ = 2.36<br>*P* = 0.114	**$F_{2, 26}$ = 6.34**<br>***P* = 0.006**	$F_{2, 26}$ = 0.36<br>*P* = 0.704	$F_{2, 24}$ = 0.61<br>*P* = 0.554	$F_{2, 75}$ = 2.77<br>*P* = 0.069	$F_{2, 26}$ = 0.32<br>*P* = 0.730	$F_{2, 26}$ = 0.43<br>*P* = 0.653	$F_{2, 26}$ = 1.15<br>*P* = 0.33	**$X^2_{2, 75}$ = 15.98**<br>***P* < 0.001**	$X^2_{2, 75}$ = 5.53<br>*P* = 0.063	**$X^2_{2, 75}$ = 27.12**<br>***P* < 0.001**
	Tsechelidis	Wind	**68.01 ±5.78(b)**	14.53 ±0.66	**40.75 ±0.94(b)**	5.59 ±0.42	1.80 ±0.10	0.90 ±0.01	4.64 ±0.22	5.27 ±0.54	3.07 ±0.13	**16.67(b)**	11.11	**72.22(a)**
		Open	**98.24 ±3.96(a)**	15.61 ±0.56	**46.59 ±1.51(a)**	5.69 ±0.30	1.80 ±0.07	0.92 ±0.01	4.52 ±0.25	6.46 ±0.67	3.26 ±0.04	**55.56(a)**	22.22	**22.22(b)**
		Supplement	**106.71 ±4.25(a)**	16.40 ±0.62	**46.50 ±1.03(a)**	5.83 ±0.30	1.82 ±0.06	0.90 ±0.01	4.78 ±0.28	5.58 ±0.68	3.10 ±0.09	**72.00(a)**	20.00	**8.00(b)**
			**$F_{2, 67}$ = 17.42**<br>***P* < 0.001**	$F_{2, 26}$ = 2.30<br>*P* = 0.120	**$F_{2, 27}$ = 7.90**<br>***P* = 0.002**	$F_{2, 27}$ = 0.12<br>*P* = 0.885	$F_{2, 26}$ = 0.03<br>*P* = 0.971	$F_{2, 69}$ = 0.57<br>*P* = 0.571	$F_{2, 27}$ = 0.28<br>*P* = 0.757	$F_{2, 27}$ = 0.28<br>*P* = 0.757	$F_{2, 21}$ = 1.23<br>*P* = 0.312	**$X^2_{2, 67}$ = 14.02**<br>***P* = 0.001**	$X^2_{2, 67}$ = 1.01<br>*P* = 0.603	**$X^2_{2, 67}$ = 21.71**<br>***P* < 0.001**
	Hayward	Wind	**60.73 ±3.50(c)**	12.71 ±0.41	51.38 ±4.40	4.74 ±0.53	1.27 ±0.05	0.85 ±0.01	3.15 ±0.20	3.14 ±0.25	3.00 ±0.04	**0.00(c)**	23.08	**76.92(a)**
		Open	**89.07 ±9.54(b)**	12.79 ±0.32	56.70 ±4.11	4.32 ±0.48	1.39 ±0.07	0.87 ±0.01	3.06 ±1.17	3.40 ±0.39	3.00 ±0.06	**43.75(b)**	6.25	**50.00(a)**
		Supplement	**133.63 ±7.95(a)**	13.60 ±0.48	53.67 ±2.44	4.60 ±0.37	1.34 ±0.06	0.86 ±0.02	3.00 ±0.13	3.30 ±0.30	3.07 ±0.03	**100(a)**	0.00	**0.00(b)**
			**$F_{2, 34}$ = 19.54**<br>***P* < 0.001**	$F_{2, 24}$ = 1.44<br>*P* = 0.258	$F_{2, 23}$ = 0.51<br>*P* = 0.606	$F_{2, 23}$ = 0.21<br>*P* = 0.812	$F_{2, 24}$ = 0.74<br>*P* = 0.487	$F_{2, 35}$ = 0.32<br>*P* = 0.727	$F_{2, 24}$ = 0.21<br>*P* = 0.810	$F_{2, 22}$ = 0.14<br>*P* = 0.869	$F_{2, 20}$ = 1.15<br>*P* = 0.337	**$X^2_{2, 36}$ = 31.49**<br>***P* < 0.001**	$X^2_{2, 36}$ = 4.27<br>*P* = 0.119	**$X^2_{2, 36}$ = 17.61**<br>***P* < 0.001**

Orchard	Variety	Pollination treatment	Weight (g)	Dry matter (%)	Flesh firmness (N)	ºBrix	Acidity	S:L	TAA	TSS	Starch	Large fruits (%)	Medium fruits (%)	Small fruits (%)
I	Hayward	Wind	**68.01 ±3.04(b)**	17.15 ±0.26	60.49 ±0.98	5.32 ±0.15	1.43 ±0.03	0.89 ±0.01	3.10 ±0.15	5.30 ±0.45	3.19 ±0.07	**6.45(b)**	16.13	**77.42(a)**
		Open	**91.71 ±2.59(a)**	17.34 ±0.32	61.00 ±1.15	5.19 ±0.20	1.50 ±0.06	0.88 ±0.01	3.28 ±0.18	5.94 ±0.29	3.08 ±0.06	**45.16(a)**	38.71	**16.13(b)**
		Supplement	**94.35 ±3.93(a)**	17.68 ±0.24	61.43 ±0.98	5.37 ±0.22	1.46 ±0.04	0.86 ±0.01	3.21 ±0.17	5.15 ±0.48	3.06 ±0.13	**50(a)**	42.31	**7.69(b)**
			**$F_{2, 86}$ = 21.19<br>P < 0.001**	$F_{2, 27}$ = 0.94<br>P = 0.402	$F_{2, 27}$ = 0.13<br>P = 0.881	$F_{2, 27}$ = 0.23<br>P = 0.795	$F_{2, 26}$ = 0.55<br>P = 0.587	$F_{2, 83}$ = 2.88<br>P = 0.062	$F_{2, 27}$ = 0.27<br>P = 0.766	$F_{2, 26}$ = 1.04<br>P = 0.369	$F_{2, 25}$ = 0.75<br>P = 0.485	**$X^2_{2, 85}$ = 18.00<br>P < 0.001**	$X^2_{2, 67}$ = 5.89<br>P = 0.053	**$X^2_{2, 67}$ = 39.59<br>P < 0.001**
K	BoErica	Wind	**86.83 ±4.81(b)**	16.46 ±0.81	48.94 ±2.59	6.91 ±0.62	1.40 ±0.10	0.88 ±0.02	2.19 ±0.12	5.23 ±0.58	2.99 ±0.04	**26.67(b)**	**46.67(a)**	26.67
		Open	**128.92 ±4.70(a)**	16.50 ±0.48	48.16 ±2.30	6.28 ±0.38	1.17 ±0.11	0.83 ±0.02	2.49 ±0.09	6.14 ±0.85	2.96 ±0.04	**81.48(a)**	**3.70(b)**	14.82
		Supplement	**137.97 ±5.02(a)**	16.73 ±0.45	50.00 ±2.21	6.64 ±0.43	1.34 ±0.06	0.85 ±0.02	2.24 ±0.13	5.99 ±0.60	3.02 ±0.04	**86.62(a)**	**7.69(b)**	7.69
			**$F_{2, 64}$ = 23.61<br>P < 0.001**	$F_{2, 23}$ = 0.07<br>P = 0.935	$F_{2, 24}$ = 0.17<br>P = 0.845	$F_{2, 24}$ = 0.45<br>P = 0.644	$F_{2, 24}$ = 1.76<br>P = 0.194	$F_{2, 64}$ = 0.44<br>P = 0.300	$F_{2, 24}$ = 2.10<br>P = 0.144	$F_{2, 23}$ = 0.41<br>P = 0.669	$F_{2, 23}$ = 0.64<br>P = 0.536	**$X^2_{2, 65}$ = 16.79<br>P < 0.001**	**$X^2_{2, 65}$ = 13.41<br>P = 0.001**	**$X^2_{2, 65}$ = 2.64<br>P = 0.267**
L	Hayward	Wind	**48.93 ±3.76(b)**	16.68 ±0.68	47.31 ±1.94	7.43 ±0.58	1.37 ±0.08	0.88 ±0.01	2.91 ±0.28	4.74 ±0.31	3.10 ±0.02	**0.00(b)**	0.00	**100.00(a)**
		Open	**62.56 ±4.05(b)**	16.15 ±0.42	47.64 ±1.60	7.52 ±0.33	1.50 ±0.07	0.87 ±0.01	2.70 ±0.36	4.97 ±0.18	3.12 ±0.02	**3.70(b)**	22.22	**74.07(a)**
		Supplement	**111.74 ±3.76(a)**	16.60 ±0.36	51.18 ±1.17	7.67 ±0.33	1.53 ±0.05	0.87 ±0.01	2.61 ±0.28	4.63 ±0.32	3.12 ±0.02	**71.43(a)**	28.57	**0.00(b)**
			**$F_{2, 60}$ = 57.01<br>P < 0.001**	$F_{2, 24}$ = 0.37<br>P = 0.689	$F_{2, 24}$ = 2.01<br>P = 0.156	$F_{2, 24}$ = 0.09<br>P = 0.913	$F_{2, 24}$ = 1.35<br>P = 0.279	$F_{2, 60}$ = 0.03<br>P = 0.968	$F_{2, 24}$ = 0.21<br>P = 0.814	$F_{2, 27}$ = 0.94<br>P = 0.404	$F_{2, 24}$ = 0.31<br>P = 0.735	**$X^2_{2, 60}$ = 38.14<br>P < 0.001**	$X^2_{2, 60}$ = 4.64<br>P = 0.099	**$X^2_{2, 60}$ = 55.65<br>P < 0.001**
M	Hayward	Open	87.66 ±4.34	16.57 ±0.41	41.49 ±2.70	7.72 ±0.34	1.31 ±0.07	0.88 ±0.01	3.37 ±0.16	3.43 ±0.26	3.09 ±0.03	35.71	28.57	35.71
		Supplement	89.59 ±4.33	17.00 ±0.43	44.40 ±1.65	7.20 ±0.40	1.31 ±0.05	0.87 ±.001	3.42 ±0.15	3.62 ±0.33	3.10 ±0.02	24.00	32.00	44.00
			$F_{1, 51}$ = 0.10<br>P = 0.755	$F_{1, 52}$ = 0.50<br>P = 0.490	$F_{1, 18}$ = 0.84<br>P = 0.371	$F_{1, 18}$ = 0.98<br>P = 0.336	$F_{1, 18}$ = 0.00<br>P = 0.971	$F_{1, 50}$ = 0.24<br>P = 0.627	$F_{1, 18}$ = 0.05<br>P = 0.828	$F_{1, 18}$ = 0.22<br>P = 0.646	$F_{1, 15}$ = 0.10<br>P = 0.758	$X^2_{1, 51}$ = 0.87<br>P = 0.352	$X^2_{1, 51}$ = 0.07<br>P = 0.786	$X^2_{1, 51}$ = 0.39<br>P = 0.540
N	Hayward	Wind	**52.31 ±1.92(b)**	13.17 ±0.75	52.90 ±1.96	3.73 ±0.27	1.47 ±0.09	0.88 ±0.03	1.79 ±0.09	3.50 ±0.52	2.52 ±0.19	**0.00(b)**	0.00	**100.00(a)**
		Open	**93.65 ±3.48(a)**	14.43 ±0.56	52.03 ±1.70	4.12 ±0.26	1.58 ±0.04	0.89 ±0.01	2.15 ±0.14	4.70 ±0.86	2.67 ±0.14	**48.28(a)**	34.48	**17.24(b)**
		Supplement	**101.08 ±2.48(a)**	14.65 ±0.45	53.84 ±1.75	4.05 ±0.17	1.49 ±0.07	0.87 ±0.02	1.98 ±0.10	5.44 ±0.87	2.38 ±0.23	**71.43(a)**	25.00	**7.14(b)**
			**$F_{2, 63}$ = 28.90<br>P < 0.001**	$F_{2, 26}$ = 1.79<br>P = 0.187	$F_{2, 25}$ = 0.47<br>P = 0.631	$F_{2, 26}$ = 0.75<br>P = 0.481	$F_{2, 26}$ = 0.80<br>P = 0.461	$F_{2, 64}$ = 0.69<br>P = 0.505	$F_{2, 25}$ = 2.57<br>P = 0.097	$F_{2, 23}$ = 1.54<br>P = 0.235	$F_{2, 25}$ = 0.56<br>P = 0.581	**$X^2_{2, 63}$ = 16.16<br>P < 0.001**	**$X^2_{2, 60}$ = 6.45<br>P = 0.040**	**$X^2_{2, 60}$ = 32.04<br>P < 0.001**
O	Hayward	Open	94.69 ±2.86	16.98 ±0.69	52.69 ±1.28	5.54 ±0.36	1.91 ±0.07	0.86 ±0.02	2.53 ±0.18	5.81 ±0.66	2.85 ±0.08	53.85	26.92	19.23
		Supplement	93.21 ±2.50	18.09 ±0.68	54.23 ±1.43	5.30 ±0.33	1.87 ±0.07	0.86 ±0.02	2.84 ±0.13	6.01 ±0.89	2.86 ±0.15	38.10	42.86	19.05
			$F_{1, 44}$ = 0.14<br>P = 0.709	$F_{1, 17}$ = 1.32<br>P = 0.267	$F_{1, 18}$ = 0.25<br>P = 0.627	$F_{1, 18}$ = 0.25<br>P = 0.626	$F_{1, 17}$ = 0.15<br>P = 0.707	$F_{1, 45}$ = 0.06<br>P = 0.802	$F_{1, 18}$ = 2.05<br>P = 0.169	$F_{1, 18}$ = 0.03<br>P = 0.859	$F_{1, 15}$ = 0.01<br>P = 0.909	$X^2_{1, 45}$ = 1.16<br>P = 0.281	$X^2_{1, 45}$ = 1.31<br>P = 0.252	$X^2_{1, 51}$ = 0.00<br>P = 0.987

Table S3. Mean values (± std error) of weight and quality parameters, and percentage of fruits in different caliber groups of kiwifruits resulting from open and supplementary pollination treatments applied in the 22 orchards surveyed in 2019. F and P values resulting from Generalized linear model analysis are shown. Statistically significant differences are highlighted in bold and significant differences between pollination treatments are indicated by different letters. Large fruits include fruits within caliber 18 to 30, Medium include fruits within caliber 33 to 36 and Small fruits includ fruits in calibers 39 to 46, class II and non-marketable fruits.

Orchard	Variety	Pollination treatment	Weight (g)	Dry matter (%)	Flesh firmness (N)	ºBrix	Acidity	S:L	Large fruits (%)	Medium fruits (%)	Small fruits (%)
A1	BoErica	Open	109.81 ±4.00	13.40 ±0.21	75.84 ±2.45	6.06 ±0.14	1.40 ±0.02	0.90 ±0.01	76.67	10.00	13.33
		Supplement	119.75 ±3.06	13.38 ±0.18	78.55 ±2.23	6.29 ±0.11	1.36 ±0.02	0.90 ±0.01	93.33	6.67	0.00
			$F_{1, 58}$ = 3.90	$F_{1, 28}$ = 0.01	$F_{1, 58}$ = 0.67	$F_{1, 58}$ = 1.18	$F_{1, 58}$ = 1.09	$F_{1, 58}$ = 0.05	$X^2_{1, 58}$ = 3.43	$X^2_{1, 58}$ = 0.22	**$X^2_{1, 58}$ = 5.83**
			P = 0.053	P = 0.923	P = 0.416	P = 0.282	P = 0.300	p = 0.816	p = 0.064	p = 0.639	**p = 0.016**
A3	*A. chinensis*	Open	92.09 ±5.26	16.28 ±0.96	49.40 ±4.31	9.24 ±0.48	1.54 ±0.08	0.86 ±0.03	33.33	33.33	33.33
		Supplement	97.03 ±3.26	16.15 ±0.60	49.79 ±1.82	9.74 ±0.30	1.53 ±0.03	0.90 ±0.01	50.00	40.00	10.00
			$F_{1, 25}$ =0.60	$F_{1, 25}$ = 0.01	$F_{1, 26}$ = 0.01	$F_{1, 24}$ = 0.77	$F_{1, 22}$ = 0.02	$F_{1, 27}$ = 0.09	$X^2_{1, 27}$ = 0.71	$X^2_{1, 27}$ = 0.12	$X^2_{1, 27}$ = 2.20
			P=0.444	P = 0.913	P = 0.924	P = 0.389	P = 0.902	P = 0.117	P = 0.400	P = 0.731	P = 0.138
B	BoErica	Open	95.36 ±3.00	13.55 ±0.49	75.67 ±1.43	5.65 ±0.15	1.47 ±0.03	0.86 ±0.01	50.00	36.67	13.33
		Supplement	93.65 ±3.36	14.06 ±0.50	79.35 ±1.49	5.50 ±0.15	1.52 ±0.03	0.88 ±0.01	36.67	46.67	16.67
			$F_{1, 56}$ = 0.14	$F_{1, 28}$ = 0.53	$F_{1, 58}$ = 3.16	$F_{1, 54}$ = 0.50	$F_{1, 58}$ =1.29	$F_{1, 57}$ = 2.66	$X^2_{1, 58}$ = 1.09	$X^2_{1, 58}$ = 0.62	$X^2_{1, 58}$ = 0.13
			P = 0.707	P = 0.472	P = 0.081	P = 0.484	P = 0.261	P = 0.109	P = 0.297	P = 0.432	P = 0.717
	Hayward	Open	95.53 ±3.77	13.60 ±0.42	69.17 ±1.81	5.06 ±0.15	1.42 ±0.03	0.83 ±0.02	32.14	28.57	39.29
		Supplement	95.11 ±3.29	14.02 ±0.23	73.50 ±2.01	4.81 ±0.13	1.47 ±0.03	0.81 ±0.02	36.00	24.00	40.00
			$F_{1, 50}$ = 0.01	$F_{1, 32}$ =0.51	$F_{1, 50}$ = 2.58	$F_{1, 49}$ = 1.49	$F_{1, 50}$ = 1.19	$F_{1, 50}$ = 0.74	$X^2_{1, 51}$ = 0.09	$X^2_{1, 51}$ = 0.14	$X^2_{1, 51}$ = 0.00
			P = 0.935	P = 0.481	P = 0.115	P = 0.228	P = 0.281	P = 0.394	P = 0.767	P = 0.706	P = 0.957
C1	Hayward	Open	116.84 ±3.71	12.33 ±0.24	69.54 ±1.32	9.64 ±0.14	1.57 ±0.04	0.92 ±0.01	90.00	6.67	3.33
		Supplement	119.49 ±3.62	12.39 ±0.23	69.95 ±1.33	9.72 ±0.17	1.53 ±0.03	0.91 ±0.01	83.33	10.00	6.67
			$F_{1,57}$ = 0.26	$F_{1,28}$ = 0.04	$F_{1,58}$ = 0.05	$F_{1,55}$ = 0.15	$F_{1,57}$ = 0.60	$F_{1,56}$ = 0.05	$X^2_{1,58}$ = 0.58	$X^2_{1,58}$ = 0.21	$X^2_{1,58}$ = 0.36
			P = 0.611	P = 0.850	P = 0.827	P = 0.700	P = 0.443	P = 0.823	P = 0.445	P = 0.639	P = 0.550
C2	Hayward	Open	107.91 ±3.63	13.04 ±0.29	61.94 ±1.55	8.31 ±0.22	1.55 ±0.03	0.90 ±0.01	72.41	13.79	13.79
		Supplement	110.93 ±3.58	12.86 ±0.39	62.44 ±1.36	8.20 ±0.20	1.53 ±0.03	0.90 ±0.01	78.57	7.14	14.29
			$F_{1,54}$ = 0.35	$F_{1,27}$ = 0.14	$F_{1,55}$ = 0.06	$F_{1,56}$ = 0.14	$F_{1,55}$ = 0.17	$F_{1,54}$ = 0.00	$X^2_{1,55}$ = 0.29	$X^2_{1,55}$ = 0.68	$X^2_{1,55}$ = 0.003
			P = 0.556	P = 0.709	P = 0.810	P = 0.709	P = 0.681	P = 0.936	P = 0.589	P = 0.409	P = 0.957
E	*A. chinensis*	Open	86.82 ±2.38	13.93 ±0.24	62.24 ±0.99	7.86 ±0.36	1.49 ±0.03	0.91 ±0.01	25.00	50.00	25.00
		Supplement	91.32 ±2.94	13.87 ±0.25	60.10 ±1.18	8.34 ±0.34	1.44 ±0.04	0.89 ±0.13	37.04	40.74	22.22
			$F_{1,53}$ = 1.43	$F_{1,53}$ = 0.03	$F_{1,53}$ = 1.94	$F_{1,53}$ = 0.94	$F_{1,53}$ = 0.71	$F_{1,53}$ = 1.24	$X^2_{1,53}$ = 0.94	$X^2_{1,53}$ = 0.48	$X^2_{1,53}$ = 0.06
			P = 0.237	P = 0.864	P = 0.170	P = 0.336	P = 0.405	P = 0.270	P = 0.333	P = 0.490	P = 0.808
F	Earlygreen	Open	80.92 ±2.94	11.37 ±0.24	78.88 ±1.54	6.19 ±0.15	1.41 ±0.04	0.91 ±0.01	11.11	40.74	48.15
		Supplement	80.32 ±3.98	11.42 ±0.20	75.59 ±1.48	6.17 ±0.14	1.40 ±0.04	0.91 ±0.01	26.92	30.77	42.31
			$F_{1,51}$ = 0.02	$F_{1,51}$ = 0.03	$F_{1,51}$ = 2.36	$F_{1,51}$ = 0.01	$F_{1,51}$ = 0.05	$F_{1,49}$ = 0.08	$X^2_{1,51}$ = 2.21	$X^2_{1,51}$ = 0.57	$X^2_{1,51}$ = 0.18
			P = 0.904	P = 0.862	P = 0.130	P = 0.925	P = 0.829	P = 0.784	P = 0.137	P = 0.449	P = 0.669
G	BoErica	Open	89.92 ±4.48	17.98 ±0.34	**93.50 ±1.58(a)**	6.91 ±0.16	1.58 ±0.03	0.90 ±0.01	39.13	8.69	52.17
		Supplement	90.67 ±4.75	18.04 ±0.36	**87.50 ±1.48(b)**	6.72 ±0.17	1.59 ±0.04	0.91 ±0.01	39.13	26.09	34.78
			$F_{1,44}$ = 0.01	$F_{1,28}$ = 0.08	**$F_{1,44}$ = 7.68**	$F_{1,44}$ = 0.67	$F_{1,44}$ = 0.03	$F_{1,44}$ = 0.08	$X^2_{1,56}$ = 0.00	$X^2_{1,56}$ = 2.51	$X^2_{1,56}$ = 1.42
			P = 0.909	P = 0.778	**P = 0.008**	P = 0.417	P = 0.872	P = 0.789	P = 1.00	P = 0.113	P = 0.233

Table S3. (Cont.)

Orchard	Variety	Pollination treatment	Weight (g)	Dry matter (%)	Flesh firmness (N)	ºBrix	Acidity	S:L	Large fruits (%)	Medium fruits (%)	Small fruits (%)
H	Earlygreen	Open	95.71 ±2.48	10.98 ±0.30	47.06 ±2.42	7.93 ±0.20	1.28 ±0.04	0.87 ±0.01	53.33	30.00	16.67
		Supplement	98.00 ±3.12	10.97 ±0.25	48.27 ±2.16	7.67 ±0.17	1.31 ±0.03	0.83 ±0.02	42.86	39.29	17.86
			$F_{1,56}$ = 0.33	$F_{1,56}$ = 0.00	$F_{1,57}$ = 0.34	$F_{1,57}$ = 0.46	$F_{1,55}$ = 0.21	$F_{1,57}$ = 3.22	$X^2_{1,51}$ = 0.85	$X^2_{1,51}$ = 0.41	$X^2_{1,51}$ = 0.16
			P = 0.568	P = 0.948	P = 0.561	P = 0.499	P = 0.647	P = 0.078	P = 0.357	P = 0.520	P = 0.692
	Tsechelidis	Open	122.50 ±4.40	15.14 ±0.24	58.19 ±1.45	4.65 ±0.11	1.78 ±0.03	0.89 ±0.01	93.10	6.90	0.00
		Supplement	123.33 ±4.71	15.17 ±0.27	57.78 ±1.52	4.65 ±0.12	1.83 ±0.04	0.90 ±0.01	85.19	7.40	7.40
			$F_{1,54}$ = 0.02	$F_{1,51}$ = 0.01	$F_{1,54}$ = 0.04	$F_{1,52}$ = 0.00	$F_{1,54}$ = 1.78	$F_{1,54}$ = 0.09	$X^2_{1,54}$ = 0.93	$X^2_{1,54}$ = 0.005	$X^2_{1,54}$ = 3.00
			P = 0.898	P = 0.931	P = 0.848	P = 0.981	P = 0.313	P = 0.765	P = 0.335	P = 0.941	P = 0.083
	Hayward	Open	126.57 ±3.21	14.59 ±0.21	69.81 ±1.35	5.76 ±0.19	1.37 ±0.02	0.88 ±0.01	90.00	3.33	6.67
		Supplement	130.42 ±3.22	14.26 ±0.27	68.85 ±1.61	5.76 ±0.16	1.37 ±0.02	0.90 ±0.01	89.66	0.00	10.34
			$F_{1,57}$ = 0.72	$F_{1,28}$ = 0.93	$F_{1,57}$ = 0.22	$F_{1,57}$ = 0.00	$F_{1,53}$ = 0.03	$F_{1,52}$ = 1.87	$X^2_{1,57}$ = 0.00	$X^2_{1,57}$ = 1.37	$X^2_{1,57}$ = 0.26
			P = 0.401	P = 0.342	P = 0.645	P = 0.994	P = 0.872	P = 0.177	P = 0.965	P = 0.242	P = 0.611
I	Hayward	Open	93.02 ±3.28	13.20 ±0.38	77.99 ±1.03	4.19 ±0.12	1.59 ±0.03	0.93 ±0.01	41.39	37.93	20.69
		Supplement	89.44 ±2.88	13.56 ±0.36	77.82 ±0.90	4.24 ±0.11	1.59 ±0.03	0.92 ±0.01	32.14	39.26	28.57
			$F_{1,54}$ = 0.66	$F_{1,28}$ = 0.50	$F_{1,54}$ = 0.02	$F_{1,55}$ = 0.09	$F_{1,55}$ = 0.00	$F_{1,53}$ = 0.27	$X^2_{1,55}$ = 0.52	$X^2_{1,55}$ = 0.01	$X^2_{1,55}$ = 0.48
			P = 0.419	P = 0.485	P = 0.900	P = 0.767	P = 0.993	P = 0.608	P = 0.469	P = 0.916	P = 0.489
J	Hayward	Open	78.45 ±5.65	15.18 ±0.42	74.31 ±1.30	7.93 ±2.27	1.39 ±0.06	0.88 ±0.02	14.26	28.57	57.14
		Supplement	86.68 ±4.72	18.84 ±0.47	72.01 ±1.88	8.00 ±0.39	1.29 ±0.05	0.92 ±0.01	25.00	37.5	37.5
			$F_{1,12}$ = 1.26	$F_{1,13}$ = 0.28	$F_{1,12}$ = 0.87	$F_{1,12}$ = 0.02	$F_{1,12}$ = 1.63	$F_{1,13}$ = 2.87	$X^2_{1,13}$ = 0.27	$X^2_{1,13}$ = 0.13	$X^2_{1,13}$ = 0.58
			P = 0.283	P = 0.608	P = 0.368	P = 0.887	P = 0.224	P = 0.114	P = 0.601	P = 0.714	P = 0.446
K	BoErica	Open	**82.86 ±3.13(b)**	13.77 ±0.33	70.09 ±1.79	5.76 ±0.15	1.62 ±0.04	0.85 ±0.02	21.43	14.29	64.29
		Supplement	**93.07 ±2.55(a)**	13.02 ±0.28	67.86 ±1.59	5.84 ±0.15	1.64 ±0.05	0.88 ±0.02	28.57	42.86	28.57
			**$F_{1,51}$ = 6.46**	$F_{1,50}$ = 2.90	$F_{1,52}$ = 0.86	$F_{1,52}$ = 0.13	$F_{1,54}$ = 0.10	$F_{1,52}$ = 1.23	$X^2_{1,54}$ = 0.38	**$X^2_{1,54}$ = 5.80**	**$X^2_{1,54}$ = 7.35**
			**P = 0.014**	P = 0.095	P = 0.358	P = 0.724	P = 0.756	P = 0.273	P = 0.537	**P = 0.016**	**P = 0.007**
L	BoErica	Open	**80.27 ±5.59(b)**	15.34 ±0.54	**81.29 ±2.24(b)**	8.39 ±0.33	1.61 ±0.04	0.91 ±0.01	25.00	31.25	43.75
		Supplement	**108.34 ±2.50(a)**	15.82 ±0.26	**88.64 ±2.21(a)**	7.98 ±0.25	1.61 ±0.02	0.92 ±0.01	86.21	13.79	0.00
			**$F_{1,43}$ = 27.71**	$F_{1,42}$ = 0.38	**$F_{1,43}$ = 4.64**	$F_{1,43}$ = 1.01	$F_{1,43}$ = 0.02	$F_{1,41}$ = 0.57	**$X^2_{1,43}$ = 17.31**	$X^2_{1,43}$ = 1.89	**$X^2_{1,43}$ = 16.97**
			**P < 0.001**	P = 0.800	**P = 0.037**	P = 0.321	P = 0.895	P = 0.454	**P < 0.001**	P =0.169	**P < 0.001**
	Hayward	Open	**93.85 ±11.54(b)**	16.69 ±0.41	62.42 ±2.23	1.63 ±0.04	8.90 ±0.39	0.91 ±0.01	54.55	0.00	45.45
		Supplement	**115.71 ±3.93(a)**	16.00 ±0.36	65.92 ±1.11	1.62 ±0.03	7.90 ±0.29	0.93 ±0.01	92.59	3.70	3.70
			**$F_{1,36}$ = 5.28**	$F_{1,17}$ = 3.53	$F_{1,35}$ = 2.47	$F_{1,36}$ = 3.73	$F_{1,36}$ = 0.00	$F_{1,36}$ = 2.17	**$X^2_{1,36}$ = 6.89**	$X^2_{1,36}$ = 0.70	**$X^2_{1,36}$ = 9.44**
			**P = 0.028**	P = 0.078	P = 0.125	P = 0.061	P = 0.952	P = 0.149	**P = 0.009**	P = 0.405	**P = 0.002**
N	Hayward	Open	**102.50 ±3.94(b)**	13.80 ±0.24	66.82 ±1.49	5.69 ±0.11	1.71 ±0.03	0.92 ±0.01	63.33	23.33	13.33
		Supplement	**116.28 ±3.45(a)**	13.65 ±0.22	70.23 ±1.04	5.56 ±0.16	1.69 ±0.03	0.89 ±0.01	95.83	4.17	0.00
			**$F_{1,54}$ = 6.40**	$F_{1,51}$ = 0.20	$F_{1,49}$ = 2.69	$F_{1,50}$ = 0.52	$F_{1,51}$ = 0.13	$F_{1,54}$ = 3.59	**$X^2_{1,52}$ = 9.47**	**$X^2_{1,52}$ = 4.39**	**$X^2_{1,52}$ = 4.96**
			**P = 0.014**	P = 0.654	P = 0.108	P = 0.473	P = 0.724	P = 0.064	**P = 0.002**	**P = 0.036**	**P = 0.026**
O	Hayward	Open	121.11 ±3.50	15.73 ±0.31	73.73 ±1.43	5.29 ±0.14	1.68 ±0.05	0.89 ±0.01	86.67	0.00	13.33
		Supplement	120.78 ±4.04	15.54 ±0.33	73.83 ±1.33	5.19 ±0.12	1.74 ±0.05	0.89 ±0.01	82.14	10.71	7.14
			$F_{1,56}$ = 0.004	$F_{1,56}$ = 0.18	$F_{1,56}$ = 0.002	$F_{1,56}$ = 0.30	$F_{1,56}$ = 0.75	$F_{1,52}$ = 0.03	$X^2_{1,56}$ = 0.22	**$X^2_{1,56}$ = 4.55**	$X^2_{1,56}$ = 0.61
			P = 0.950	P = 0.671	P = 0.961	P = 0.586	P = 0.389	P = 0.867	P = 0.634	**P = 0.033**	P = 0.435

Table S3. (Cont.)

Orchard	Variety	Pollination treatment	Weight (g)	Dry matter (%)	Flesh firmness (N)	ºBrix	Acidity	S:L	Large fruits (%)	Medium fruits (%)	Small fruits (%)
P	Hayward	Open	111.55 ±3.67	15.79 ±0.27	68.53 ±1.38	7.36 ±0.18	1.80 ±0.03	0.84 ±0.01	70.00	13.33	16.67
		Supplement	122.87 ±4.38	16.03 ±0.33	69.16 ±1.44	7.31 ±0.20	1.77 ±0.04	0.82 ±0.02	74.07	11.11	14.81
			$F_{1,55} = 3.97$	$F_{1,28} = 0.32$	$F_{1,55} = 0.10$	$F_{1,54} = 0.02$	$F_{1,55} = 0.40$	$F_{1,55} = 1.19$	$X^2_{1,55} = 0.12$	$X^2_{1,55} = 0.07$	$X^2_{1,55} = 0.04$
			$P = 0.051$	$P = 0.575$	$P = 0.753$	$P = 0.878$	$P = 0.531$	$P = 0.281$	$P = 0.732$	$P = 0.798$	$P = 0.848$
Q	Hayward	Open	103.69 ±3.42	13.01 ±0.28	71.48 ±1.24	7.52 ±0.19	1.52 ±0.04	0.88 ±0.01	58.62	24.14	17.24
		Supplement	103.73 ±2.81	12.99 ±0.27	69.71 ±1.02	7.93 ±0.20	1.51 ±0.03	0.89 ±0.01	78.57	14.29	7.14
			$F_{1,55} = 0.00$	$F_{1,55} = 0.00$	$F_{1,55} = 1.19$	$F_{1,52} = 2.26$	$F_{1,55} = 0.05$	$F_{1,50} = 0.18$	$X^2_{1,55} = 2.66$	$X^2_{1,55} = 0.90$	$X^2_{1,53} = 1.39$
			$P = 0.993$	$P = 0.969$	$P = 0.280$	$P = 0.139$	$P = 0.824$	$P = 0.670$	$P = 0.103$	$P = 0.343$	$P = 0.238$
R	Soreli	Open	88.65 ±3.75	11.92 ±0.22	55.26 ±1.08	7.95 ±0.15	1.32 ±0.03	0.97 ±0.00	39.29	28.57	32.14
		Supplement	82.13 ±3.99	11.35 ±0.27	54.74 ±1.25	7.95 ±0.22	1.31 ±0.04	0.97 ±0.00	28.00	36.00	36.00
			$F_{1,53} = 1.43$	$F_{1,51} = 2.65$	$F_{1,51} = 0.09$	$F_{1,48} = 0.00$	$F_{1,53} = 0.08$	$F_{1,49} = 0.12$	$X^2_{1,51} = 0.76$	$X^2_{1,51} = 0.33$	$X^2_{1,51} = 0.09$
			$P = 0.238$	$P = 0.109$	$P = 0.769$	$P = 0.987$	$P = 0.779$	$P = 0.736$	$P = 0.385$	$P = 0.563$	$P = 0.767$
S	Hayward	Open	161.30 ±5.49	15.76 ±0.25	69.66 ±1.37	7.30 ±0.18	1.58 ±0.04	**0.91 ±0.01(a)**	96.43	3.57	0.00
		Supplement	172.01 ±5.41	16.09 ±0.22	68.71 ±1.15	7.02 ±0.19	1.64 ±0.03	**0.88 ±0.01(b)**	96.43	0.00	3.57
			$F_{1,54} = 1.93$	$F_{1,27} = 0.97$	$F_{1,52} = 0.28$	$F_{1,52} = 1.17$	$F_{1,54} = 1.79$	$\mathbf{F_{1,53} = 7.94}$	$X^2_{1,54} = 0.00$	$X^2_{1,54} = 1.40$	$X^2_{1,54} = 1.40$
			$P = 0.171$	$P = 0.332$	$P = 0.598$	$P = 0.284$	$P = 0.187$	$\mathbf{P = 0.007}$	$P = 1.00$	$P = 0.236$	$P = 0.236$
T	Hayward	Open	**52.23 ±3.70(b)**	15.65 ±0.42	81.41 ±1.26	5.05 ±0.12	1.46 ±0.03	0.92 ±0.01	0.00	11.54	88.46
		Supplement	**73.41 ±4.05(a)**	15.53 ±0.51	84.19 ±1.25	5.19 ±0.13	1.54 ±0.03	0.92 ±0.01	4.00	32.00	64.00
			$\mathbf{F_{1,49} = 14.98}$	$F_{1,27} = 0.03$	$F_{1,49} = 2.45$	$F_{1,49} = 0.63$	$F_{1,47} = 3.48$	$F_{1,45} = 0.05$	$X^2_{1,49} = 1.45$	$X^2_{1,49} = 3.24$	$\mathbf{X^2_{1,49} = 4.38}$
			$\mathbf{P < 0.001}$	$P = 0.862$	$P = 0.124$	$P = 0.433$	$P = 0.068$	$P = 0.824$	$P = 0.229$	$P = 0.072$	$\mathbf{P = 0.036}$
U	Hayward	Open	101.09 ±2.89	15.26 ±0.22	73.97 ±1.13	4.45 ±0.08	1.47 ±0.02	0.89 ±0.01	65.51	31.03	88.46
		Supplement	103.30 ±3.05	15.29 ±0.21	75.60 ±1.05	4.65 ±0.09	1.47 ±0.02	0.90 ±0.01	57.14	35.71	7.14
			$F_{1,55} = 0.28$	$F_{1,27} = 0.01$	$F_{1,55} = 1.12$	$F_{1,55} = 2.64$	$F_{1,55} = 0.01$	$F_{1,54} = 0.298$	$X^2_{1,55} = 0.42$	$X^2_{1,55} = 0.14$	$X^2_{1,55} = 0.40$
			$P = 0.601$	$P = 0.916$	$P = 0.294$	$P = 0.110$	$P = 0.922$	$P = 0.587$	$P = 0.516$	$P = 0.708$	$P = 0.529$
V	Hayward	Open	**81.97 ±5.16(b)**	13.91 ±0.31	79.41 ±1.44	5.46 ±0.21	1.81 ±0.03	0.90 ±0.01	36.67	16.67	46.67
		Supplement	**113.29 ±3.81(a)**	14.34 ±0.31	81.68 ±1.28	5.42 ±0.19	1.81 ±0.02	0.91 ±0.01	92.86	3.57	3.57
			$\mathbf{F_{1,58} = 23.31}$	$F_{1,28} = 0.97$	$F_{1,54} = 1.37$	$F_{1,58} = 0.02$	$F_{1,56} = 0.01$	$F_{1,56} = 0.64$	$\mathbf{X^2_{1,56} = 22.09}$	$X^2_{1,56} = 2.92$	$\mathbf{X^2_{1,56} = 16.22}$
			$\mathbf{P < 0.001}$	$P = 0.334$	$P = 0.247$	$P = 0.894$	$P = 0.922$	$P = 0.426$	$\mathbf{P < 0.001}$	$P = 0.088$	$\mathbf{P < 0.001}$

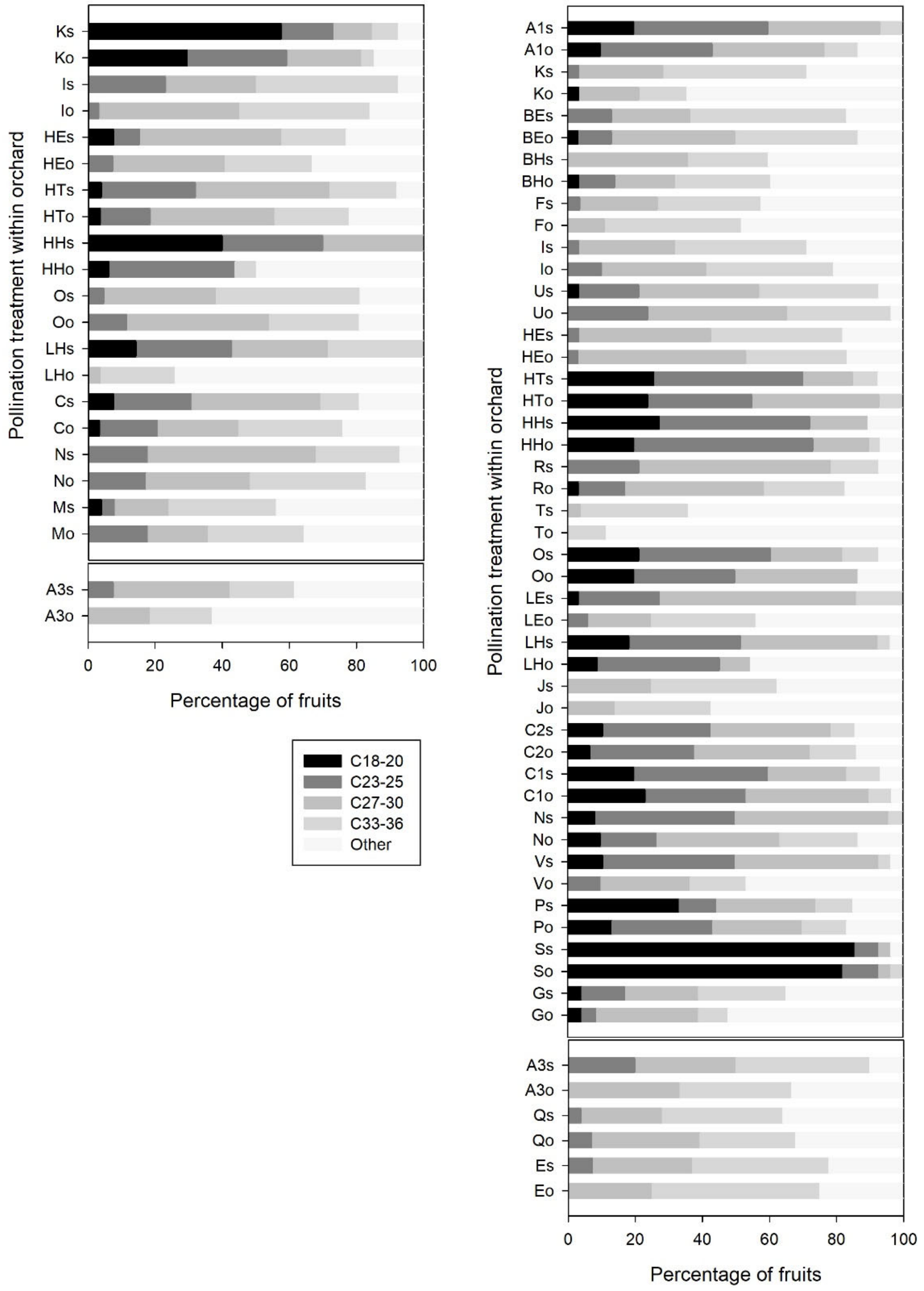

Figure S1. Distribution of kiwifruits resulting from open (O) and supplementary (S) pollination by caliber category for each orchard and variety within the 9 orchards surveyed in 2018 (left) and the 22 orchards surveyed in 2019 (right).

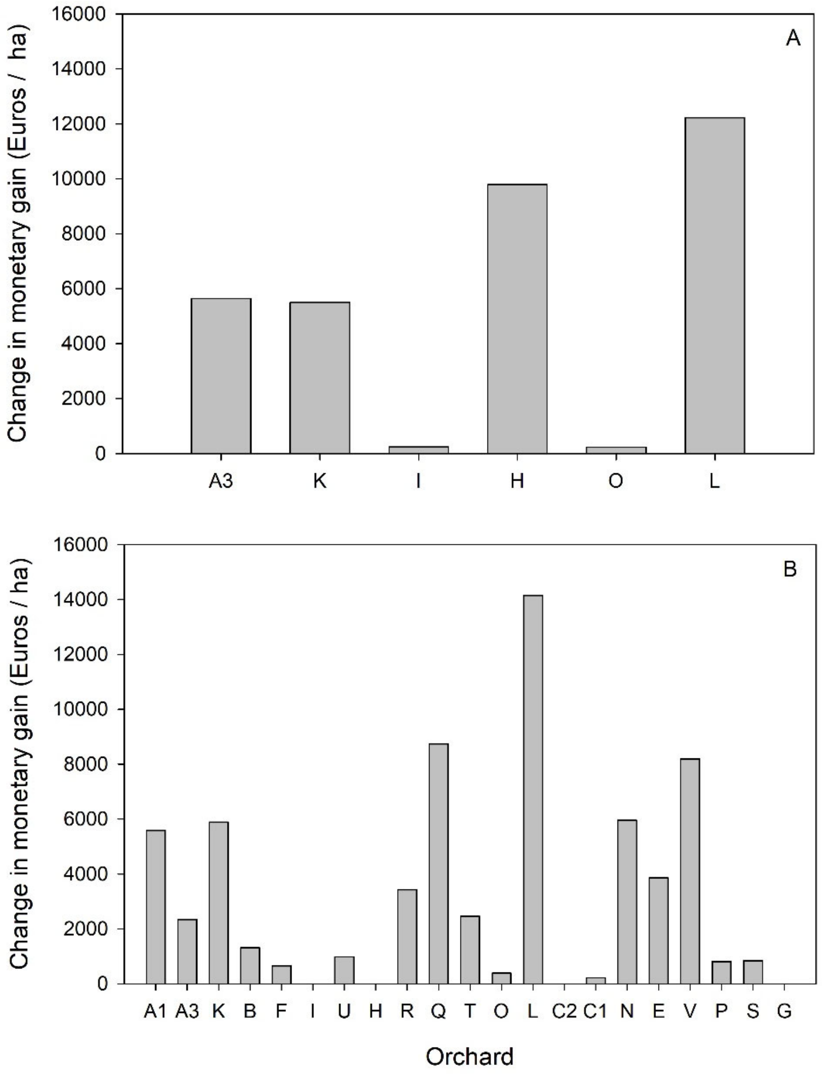

Figure S2. Change in monetary gain (Euros/ha) for the 9 orchards surveyed in 2018 (A) and the 22 orchards surveyed in 2019 (B).

Table S4. Details of kiwifruit orchards used in this study, namely, species, female and male varieties planted, male:female ratio within the orchard, age given in number of year after the orchard installation, area of the orchard in hectares, indication if the orchard is covered by hail net, plant training system, number of beehives installed per orchard , and sampling year.

Orchard	Species	♀ Variety	♂ Variety	♂:♀ ratio	Age	Area (ha)	Covered	Training system	No. Beehives	Sampling year
A1	*A. deliciosa*	BoErica	Tomuri-P1; Chieftain	1:5	11	28	No	Pergola	120	2019
A3	*A. chinensis*	unkown	Unkown	1:5	11	2.5	Yes	Pergola	45	2018, 2019
B	*A. deliciosa*	BoErica<br>Hayward	Chieftain<br>Tomuri	1:7	8<br>8	4	No	Pergola	0<br>0	2019
C1	*A. deliciosa*	Hayward	Tomuri; Matua	1:5	18	3.33	No	T-bar	0	2019
C2	*A. deliciosa*	Hayward	Tomuri; Matua	1:5	18	0.45	No	T-bar	0	2018, 2019
E	*A. chinensis*	unkown	Unkown	1:5	29	0.91	No	Pergola	0	2019
F	*A. deliciosa*	Earlygreen	Tomuri	1:7	16	10	No	Pergola	0	2019
G	*A. deliciosa*	BoErica	Tomuri-P1	1:3	2	6.85	No	Pergola	0	2019
H	*A. deliciosa*	Earlygreen<br>Tsechelidis<br>Hayward	Tomuri-P1; Chieftain	1:6	10	12	No	Pergola	40	2018, 2019
I	*A. deliciosa*	Hayward	Matua	1:4	5	2	No	Pergola	0	2018, 2019
J	*A. deliciosa*	Hayward	Tomuri	1:4	5	0.89	No	T-bbar	0	2019
K	*A. deliciosa*	BoErica	Tomuri; Chieftain	1:7	9	10	No	Pergola	0	2018, 2019
L	*A. deliciosa*	BoErica<br>Hayward	Tomuri	1:4	7	3	Yes	Pergola	0	2019<br>2018, 2019
M	*A. deliciosa*	Hayward	Tomuri; Matua	1:5	30	0.5	No	T-bar	0	2018
N	*A. deliciosa*	Hayward	Tomuri; Matua	1:5	31	5	No	T-bar	0	2018, 2019
O	*A. deliciosa*	Hayward	Tomuri; Matua	1:5	7	27	No	Pergola	0	2018,2019
P	*A. deliciosa*	Hayward	Tomuri; Matua	1:5	5	2.27	No	T-bar	0	2019
Q	*A. deliciosa*	Hayward	Tomuri; Chieftain	1:5	8	6.64	No	Pergola	0	2019
R	*A. chinensis*	Soreli	Belen	1:4	8	1.86	No	Pergola	0	2019
S	*A. deliciosa*	Hayward	Tomuri; Matua	1:6	9	10	No	Pergola	0	2019
T	*A. deliciosa*	Hayward	Tomuri	1:5	7	4	No	Pergola	0	2019
U	*A. deliciosa*	Hayward	Tomuri; Matua	1:5	8	3	No	T-bar	0	2019
V	*A. deliciosa*	Hayward	Tomuri; Chieftain	1:4	28	3	No	T-bar	0	2019

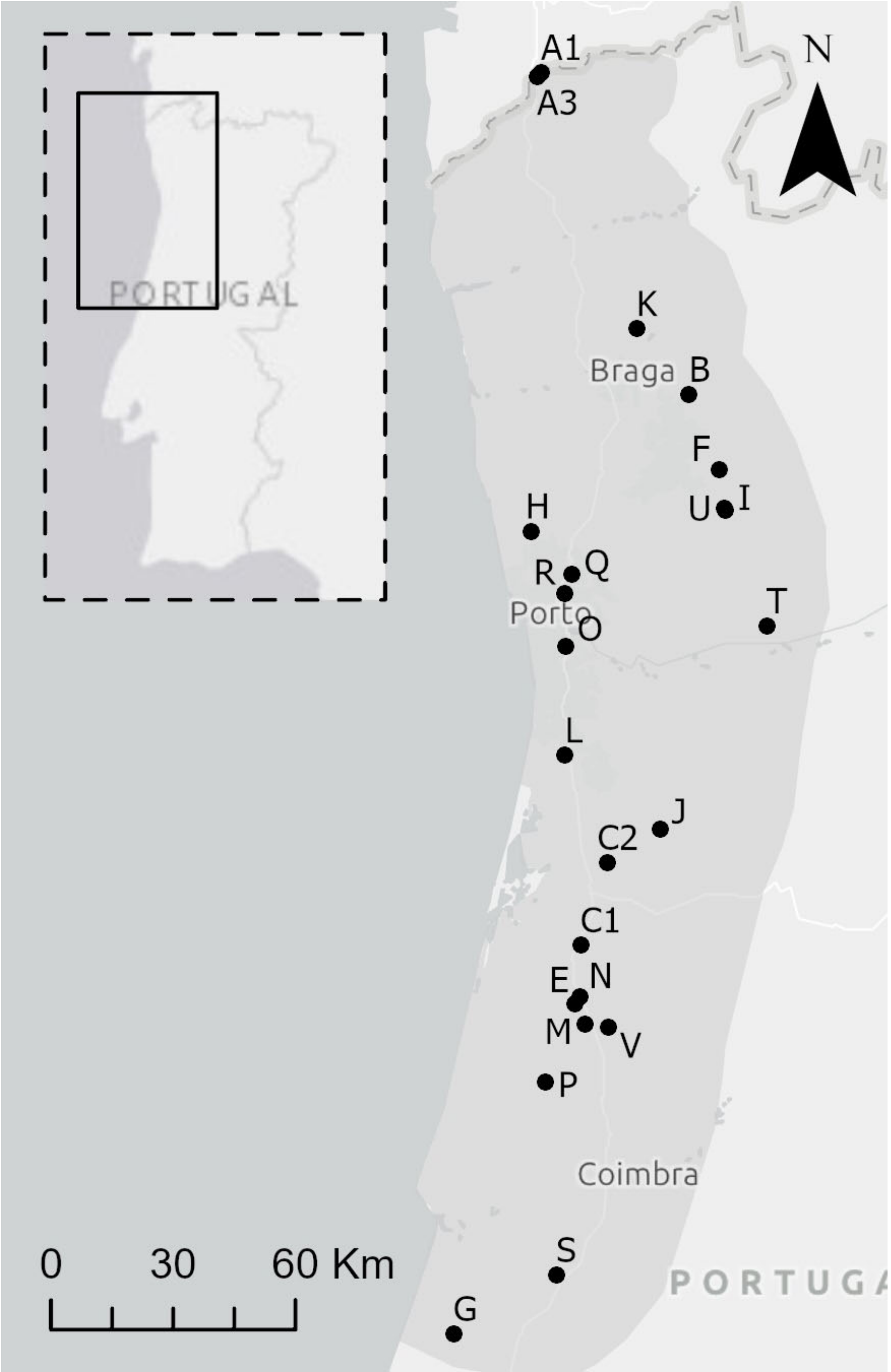

Figure S3. Distribution of sampling orchards and kiwifruit production area in Portugal (dark grey shade). The kiwifruit production area in Portugal is currently around 2,736 ha, with the main production areas located in the North (70%) and Centre (29%) of the country (INE, 2019).
